# Equal in death: Ancient genomic analysis of children’s early Christian burials

**DOI:** 10.1126/sciadv.aeb8588

**Published:** 2026-07-10

**Authors:** Maja Krzewińska, Anna Kjellström, Reyhan Yaka, Ricardo Rodríguez-Varela, Zoé Pochon, Vendela Kempe Lagerholm, Charlotte Hedenstierna-Jonson, Torun Zachrisson, Natalija Kashuba, Verónica Sobrado, Thijessen Naidoo, Kıvılcım Başak Vural, Mattias Jakobsson, Gülşah Merve Kılınç, Jan Storå, Anders Götherström

**Affiliations:** ^1^ Department of Archaeology and Classical Studies, Stockholm University, Stockholm, Sweden.; ^2^ Centre for Palaeogenetics, 106 91 Stockholm, Sweden.; ^3^ Department of Archaeology and Ancient History, Archaeology, Uppsala University, 752 38 Uppsala, Sweden.; ^4^ Stiftelsen Upplandsmuseet/County Museum of Uppland, 753 10 Uppsala, Sweden.; ^5^ Department of Organismal Biology, Human Evolution, and SciLifeLab, Uppsala University, 752 36 Uppsala, Sweden.; ^6^ Ancient DNA Unit, Science for Life Laboratory, Stockholm, Sweden.; ^7^ Department of Biological Sciences, Middle East Technical University, Ankara, Turkey.

## Abstract

Sexing the skeletal remains of young individuals is crucial yet notoriously difficult in archaeology. Children, who cannot be reliably sexed morphologically, are often excluded from gender-related research, limiting our understanding of past childhood. This issue is compounded in contexts lacking grave goods, such as early Christian burials. We conducted genomic screening of 142 individuals from Sweden dating from the late Viking Age to the Medieval period, including 68 subadults and 74 adults from 27 single and 50 multiple burials. To investigate the treatment of children in death and the role of collective graves, we applied genomic sexing and kinship analyses to individuals from three sites. Contrary to the assumption that collective burials reflect close kinship, our results show that children interred with adults rarely shared close biological ties. Burial patterns indicate that gender roles were established early, with both boys and most girls mirroring adult spatial patterns. However, flexibility existed, and extended kinship likely played a central role in structuring these communities.

## Teaser

Ancient genomes challenge views of close kinship in multiple Christian burials, revealing flexible traditions around children.

## INTRODUCTION

Childhood archaeology is a relatively new field of study. Here, we leverage ancient DNA genomics to investigate the treatment of children in early Christian burial contexts in Sweden. Using genomics to this end is particularly practical, as determining the biological sex below the age of 15 (hereafter referred to as “subadult individuals”) based on skeletal morphology of individuals presents a challenge, which is further affected by the often fragmentary nature of the remains. The “subadult” category usually follows standard osteological age classifications, consisting of fetus (prenatal), infants I (0 to 6 years), infants II (7 to 12 years), and juvenile (13 to 15 years) (*1*–*3*). Despite numerous attempts to develop methods for sex assessment based on morphology for juvenile individuals [e.g., (*4*–*7*)], developmental nuances, along with genetic and environmental factors, make osteological sexing unreliable (refer to section S1.1 for details). Consequently, reliance on external factors such as grave goods to differentiate between boys and girls has been common [e.g., (*8*, *9*)]. However, while useful in the absence of reliable assessment methods for sexing, this approach has limited power if the focus is on biological sex. Gender does not always align with biological sex, especially if the material culture is used as an inflexible gender marker, potentially obscuring insights into the social structures of past communities and, thus, the socialization of young individuals. Moreover, Christian burials often lack grave goods, necessitating direct sex identification through genetic sex identification (*10*, *11*) or nanoflow liquid chromatography mass spectrometry of sex chromosome–linked amelogenin protein isoforms (*12*).

This study examines children recovered from medieval multiple burials in Sweden to gain insight into how they were viewed and treated by contemporary society, at least in death. Notably, written sources on regulations for funerals or mortuary practices from the Viking Age in Scandinavia are scarce (*13*). The period was characterized by a large diversity in burial expression including that of form (funeral pyres or inhumations), excess, procedure, location, the size of aggregation/cemetery [e.g., (*14*)]. Most graves contained personal items, and a few were magnificently furnished. Graves were usually meant for one person and in multiple burials; one of the individuals has sometimes been interpreted as a person of lower social status (*15*, *16*). Christianity’s spread across Scandinavia in the late 10th and 11th centuries led to more uniform mortuary practices. Initially, people were inhumed at their homesteads or in reused older burial mounds (*17*, *18*). The funeral act probably did not differ from the liturgical practice in Christian Europe (*19*). Only the baptized individuals were allowed to be buried on the consecrated ground, thus excluding hastily deceased infants (*19*, *20*). Christian graves of the time were single east-west oriented burials, with a supine body lacking grave goods except for items related to the dress or shroud of the diseased and with simple or no markings above ground [e.g., (*18*, *21*)]. The preparation of the grave and body in medieval times was related to the importance of resurrection (*22*). However, some theologians already in the 9th century considered the burial procedures foremost acts of comfort for relatives (*19*).

The location of graves in the cemeteries was also linked to liturgic and ideological principles, reflecting ecclesiastical injunctions and the secular social order of the time (*19*, *23*). A social zoning system existed whereby mainly clergy, but also the lay elite, were buried near the church altar, and the burials of people of lower social status were placed in descending order farther from the spiritual center in the churchyard. The Norwegian Borgarthing’s law, for instance, states that slaves were to be buried on the outskirts of the churchyard (*24*). In addition, the Eidsivathing law (11th to 12th century) advocated that the burials were to be segregated by sex with women buried to the north and men to the south (*20*). Churchyards validating these laws are found in all Scandinavian countries [e.g., (*20*, *23*, *25*–*27*)], even if there apparently were regional differences in the implementation of sex segregation. Some scholars argue that the separation of sexes enforced by the Eidsivathing law is an anomaly, as it contradicts canon law (*28*, *29*). The Christian ideal was for married couples to rest in family tombs, near ancestors, and nonadult children, although not necessarily in the same structure. Concentrations of child burials at many churchyards additionally imply that social factors related to age could govern the location of a grave (section S1.2).

Deviations from the established practices and norms (as in the case of multiple burials) highlight subtle details reflecting individual and social attributes concerning the inhumed individual’s surroundings. These burials are present throughout medieval Scandinavia (*30*). A multiple burial (also known as plural or collective burial) is here defined as a funerary structure where two or more individuals were inhumed. There are many types and subcategories of such burials, e.g., mass graves or family tombs (section S1.3). Commonly, when no clear context for multiple inhumation is available, kinship is assumed to be the grounds for this funerary practice (*31*, *32*). While kinship is often described as biological affinity, it may also represent alternative and complex notions of social affinity, including marriage, adoption, emancipation, fostering, apprenticeship, etc. The distinction between these two main types of relations can be difficult to determine from the archaeological record (*33*). In the absence of contemporary records, the relationship between individuals sharing the same grave is difficult to ascertain. However, there are some fourth century texts in which it is assumed that married couples are buried together [cited literature in (*19*)]. A great variation in burials, such as those of children buried with men or same sex adult burials, is also common in medieval Scandinavian multiple burials (*30*, *34*), which further emphasizes the complexity of social relations untraceable in archaeological record. Therefore, the ability to exclude biological kinship between coburied individuals helps to emphasize alternative forms of social affiliations (section S1.4).

Here, we screened 142 new ancient human genomes from Sweden, including 66 subadults interred in 50 multiple burials. We selected Late Viking Age to early Christian individuals from three different locations known to include such burials, i.e., Fjälkinge (*n* = 11), Sigtuna (*n* = 50), and Västerhus (*n* = 81) [e.g., (*27*, *31*)]. We used biological sex identification, kinship analyses, karyotype, and identical-by-descent (IBD) sharing to address questions regarding treatment of children in the Viking Age and Medieval period in Sweden.

## RESULTS

### Individual and population genetic analyses

**Fig. 1.**
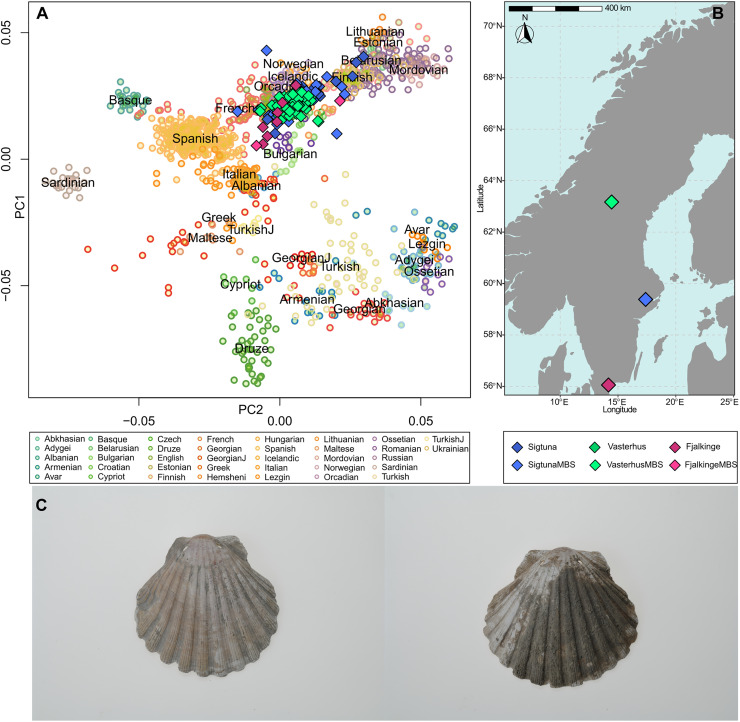
Genetic structure and archaeological context. (**A**) Genetic data from analyzed individuals projected onto the first two principal components of modern West Eurasians. (**B**) Map of Scandinavian peninsula with marked locations of sites. (**C**) The two scallop shells found at the Västerhus cemetery (wes056 and wes007) (Photo: C. Åhlin, 2012, Historiska museet. CC BY 4.0.).

We conducted genomic screening of 142 individuals from three sites in Sweden (Fig. 1). Of these, 116 individuals were recovered from 50 multiple burials, while 25 individuals from single burials served as a comparison group (referred to as baseline reference material). Two individuals were originally interred in multiple burials (mbs151 and mbv240), but as their coburied counterparts did not yield genomic data, they were treated as single-burial individuals (total *n* = 27). One individual from a multiple burial had been previously published (wes007) (*35*).

Among the 115 newly sequenced individuals from multiple burials, 21 yielded insufficient genomic coverage for kinship analysis (37,350 to 428,051 human reads; mean, 182,060). These individuals were used only for basic burial statistics (sex distribution and/or burial age composition). Mitochondrial contamination was assessed using two measures (*36*, *37*). Individuals showing >5% contamination in both tests were the same individuals with coverage <0.01×, suggesting that elevated estimates were due to low coverage; these individuals were excluded from genomic analyses. For males, X chromosome–based contamination estimates were additionally obtained (*38*). All quality assessment measures, including contamination, read length, and postmortem damage, are listed in table S2.

We prepared two datasets for downstream analyses: (i) Burial composition (sex and age) analyses used a dataset of individuals from multiple burials (*n* = 118), encompassing individuals from 51 multiple burials, including a previously published individual from Västerhus (*n* = 1) (*35*) and the two additional individuals from a single grave from Lund Cathedral (*n* = 2) (*39*, *40*). (ii) Kinship and population genomic analyses used a dataset of 144 individuals from both multiple and single burials, composed of newly sequenced genomes (coverage, >0.01× to 19×; *n* = 121) and reference genomes published elsewhere (*n* = 23) (*35*, *39*–*41*).

### Sex and age association with burial composition and location

First, we evaluated individual sex and age distributions in all multiple burials. A total of 118 individuals from 51 burials were represented by 44.9% biological females (*n* = 53), 50.0% biological males (*n* = 59), and 5.1% individuals of unassigned sex (*n* = 6). Multiple burials comprised 23.5% female-only (“same sex,” ss; *n* = 12), 31.4% male-only (*n* = 16), 35.3% mixed-sex (“mixed sex,” ms; *n* = 18), and 9.8% uncategorized (“undetermined,” na; *n* = 5) burial groups.

**Fig. 2.**
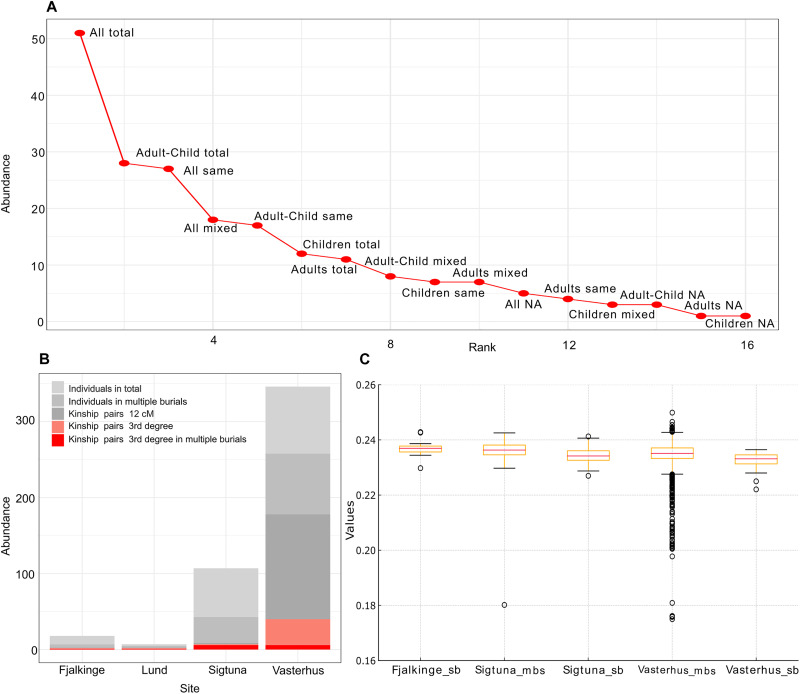
Composition of individual and multiple burials at three different sites. (**A**) Rank Abundance Plot visualizing distribution of age and sex combination in various burial types. (**B**) Abundance distribution by site and burial category dependent on identified kinship relation. (**C**) Box plot visualizing pairwise nucleotide diversity between pairs of individuals deposited in multiple burials (mbs) and single burials (sb) at the three different sites. The median values are Fjalkinge_sb: 0.236959, Sigtuna_mbs: 0.236323, Sigtuna_sb: 0.234175, Vasterhus_mbs: 0.235120, and Vasterhus_sb: 0.233146 (the Fjalkinge_mbs were not included because of low number of pairwise overlapping SNPs between individuals in the multiple burial group).

Age structures (table S3) showed that most multiple burials contained both adults and children (Adult-Child; *n* = 28, 54.9%), followed by child-only (Children; *n* = 12, 23.5%) and adult-only (Adults; *n* = 11, 21.6%) burials. When considering all combinations of age and sex, we observed an apparent overrepresentation of same-sex adult-child burials, i.e., adult men or women buried with children of the same sex (Table 1 and Fig. 2A). We conducted a Monte Carlo simulation in R, modeling expectations for nine possible burial types (Adult-Child_ss, Adult-Child_ms, Adult-Child_na, Adults_ss, Adults_ms, Adults_na, Children_ss, Children_ms, and Children_na) over 100,000 iterations. The simulations confirmed that observing 17 same-sex adult-child burials is highly unlikely by chance (*P* = 0.0002). However, a Fisher’s exact test applied to the global contingency table of burial age category (Adult-Child versus adult-only versus child-only) and sex composition (same sex versus mixed sex) found no statistically significant association (Fisher’s exact test, *P* = 0.4456). This suggests that while the same-sex adult-child pairing was the most frequent practice, the choice between same-sex or mixed-sex groupings was not strictly dictated by the age categories present.

**Table 1.** Summary burial statistics. Sex and age distribution in all burials with available data from more than one individual. The table includes the previously published Winstrup burial (*39*, *40*). No significant association was found between burial type and sex (Fisher’s exact test, *P* = 0.4456).

	Same sex	Mixed sex	Missing sex	Total
Adult-Child	17(60.7%)	8 (28.6%)	3 (10.7%)	28 (100%)
Adults	4 (33.3%)	7 (58.3%)	1 (8.3%)	12 (100%)
Children	7 (63.6%)	3 (27.3%)	1 (9.1%)	11 (100%)
Total	28	18	5	51

To evaluate whether finer age distinctions among subadults affected burial composition, we expanded the original age categorization by subdividing children into fetuses, infants (≤1 year), and children (>1 to 17 years) (table S3). Further subdivision was not feasible because of limited sample sizes. This refined analysis demonstrates that multiple burials containing adults with infants (19.6%, *n* = 10) or children (21.6%, *n* = 11) occur at frequencies comparable to adult-only groups (21.6%, *n* = 11) and subadult-only multiple burials considered collectively (23.5%, *n* = 12). These findings suggest that the inclusion of these more specific demographics does not substantially alter the broader burial composition patterns identified in the primary analysis.

**Fig. 3.**
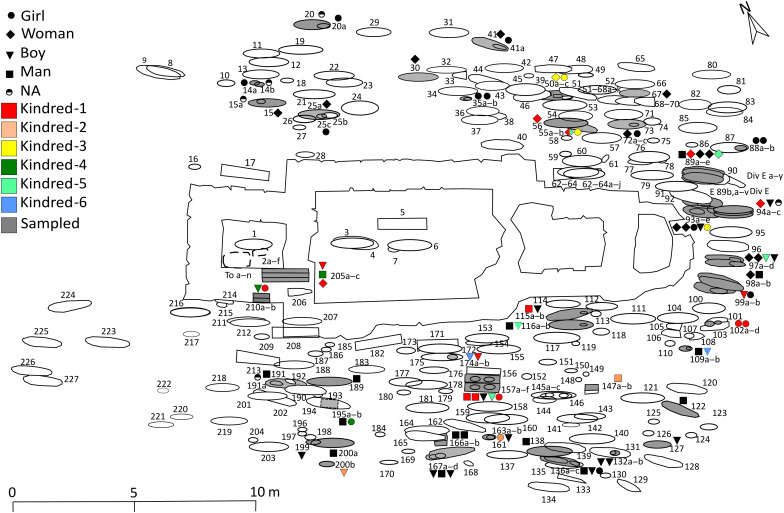
Sex, age, and kin distribution throughout the Västerhus cemetery. Kin groups were defined on the basis of up to second-degree kinship groupings, while possible reconstructed pedigrees were visualized in fig. S2. Burial plan was generated based on the work of Gejvall (*25*).

Because early Christian burial practice in parts of Scandinavia involved spatial segregation of adult men and women, we assessed the spatial patterning of subadults. In Västerhus, boys and girls broadly followed the adult spatial division. Mixed-sex burials clustered mainly on the east or west sides of the church, bypassing the north-south segregation rule. A small number of girls were buried atypically on the southern side (*n* = 4), together with adult males and boys (Fig. 3).

In Sigtuna, it is not clear whether the spatial distribution follows the same rules as in Västerhus, as the churchyards have not been fully excavated. Among all Sigtuna cemeteries, only one (Götes mack) seems to follow a pattern where women appear to cluster in the northeast and men in the southwest (*14*). However, the multiple and single burials from Sigtuna examined here originate from the southern/southeastern (Church 2, Church 3, and St. Lars’) or southwestern (St. Olaf’s) areas of the town’s burial grounds (*42*); these samples show a slight male predominance but no strong sex segregation. In Viking Age Fjälkinge, no spatial sex segregation was evident (*43*).

### Genetic relatedness and genomic diversity

To examine whether multiple burials reflected reduced genetic diversity expected from close kinship, we calculated pairwise nucleotide diversity between pairs of individuals deposited in multiple burials and single burials at the three different locations (Fig. 2C). Here, we use singly buried individuals to test whether joint interments could potentially reflect closer than random biological kinship at each of the tested sites. We find that pairwise diversity of individuals in multiple burials is on par or higher than that among individuals from single burials from the same sites, indicating that multiple burials did not predominantly consist of close kin, as this would have resulted in lower genetic diversity among coburied individuals.

We then inferred genomic kinship using Relationship Estimation from Ancient DNA (READ) (*44*), lcMLkin (*45*), and NgsRelate v2 (*46*, *47*), merging genotype data with transversions from the Estonian Genome Diversity Project (EGDP) (*48*). Because these methods rely on different statistical frameworks, their kinship estimates were not always fully concordant. We therefore adopted a conservative overlap-based approach, using only relationships identified by all three methods—together with uniparental markers and age-at-death estimates—for pedigree reconstruction. Relationships supported by fewer than three methods were considered uncertain and excluded from pedigree reconstruction. To ensure robust kinship inference, we used individuals from single interments as a baseline reference group, as they were the least likely to be closely related and therefore reduced the risk of inflating relatedness estimates among coburied individuals. We identified 45 related pairs, forming several extended kindreds (Fig. 3, figs. S1 and S2, and tables S5 to S10). We identify kindred as a group of individuals connected through biologically inferred relatedness based on genomic data. This includes first- to third-degree relationships (e.g., parent-child, siblings, grandparents, aunts/uncles, and cousins). The term is used strictly in a genetic context and does not imply any specific social, cultural, or household affiliations.

Kinship analyses revealed that, among multiple burials with sufficient genomic data from at least two individuals, only 12.0% of multiple burials at Västerhus (*n* = 25) contained first-degree relatives (*n* = 3; mbv172-mbv173, sisters; mbv250-mbv251, father-son; mbv330-mbv332, probable siblings), while no second-degree relationships were found within the same burial, although these relationships were common across burials. In Sigtuna, intraburial kinship was identified in 12.5% (*n* = 2) of the multiple burials with sufficient genomic data from at least two individuals (*n* = 16), comprising an unspecified second-degree relation (mbs021-mbs022) and a probable parent-offspring pair (mbs161-mbs162). In Fjälkinge, the only identified kinship pair (fjk531-fjk533) was not placed in the same burial and was identified as either identical twins or the same individual. As we could not exclude the possibility of osteological misidentification, the lower-coverage sample (fjk533) was excluded from all genomic analyses.

**Fig. 4.**
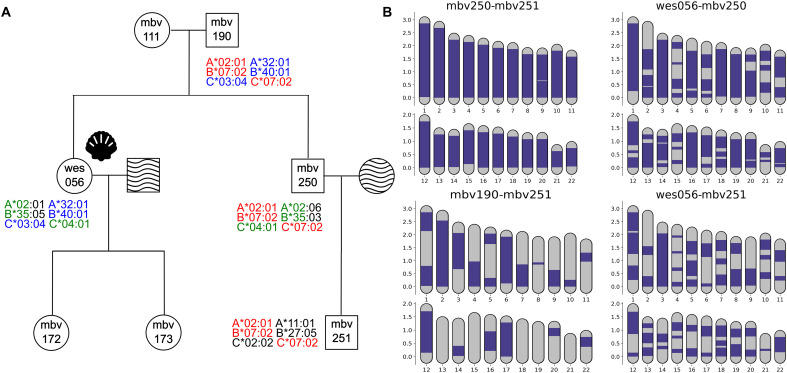
Haplotype reconstruction for core individuals in kindred-1. (**A**) HLA haplotypes in a single family group from Västerhus. On the basis of the pedigree information, it was possible to phase the results from the nf-core/HLAtyping pipline (v.1.2.0) utilizing OptiType (v.1.3.1), reconstructing the following HLA class I haplotypes: A*02:01-B*07:02-C*07:02 (mbv190, mbv250, and mbv251), A*32:01-B*40:01-C*03:04 (mbv190 and wes056), A*11:01-B*27:05-C*02:02 (mbv251), and A*02:01/02:06-B*35:03/35:05-C*04:01 (wes056, mbv250, and likely mbv111) (fig. S3). (**B**) AncIBD inferred IBD segments between selected pairs of individuals from kindred-1, where mbv250-mbv251 and wes056-mbv250 are different types of first-degree kinship (parent-offspring versus full siblings), and mbv190-mbv251 and wes056-mbv251 are different types of second-degree kinship (grandparent-grandchild versus avuncular).

Beyond broad genomic kinship, we analyzed immunogenetic diversity by reconstructing HLA (human leukocyte antigen) alleles and haplotypes within a single robustly identified family unit from Västerhus. For five members of kindred-1 (mbv111, mbv190, mbv250, mbv251, and wes056), we obtained enough additional genome coverage to successfully employ nf-core/HLAtyping pipeline (*49*) to assign HLA complex class I alleles (HLA-A, HLA-B, and HLA-C). These alleles, in combination with kinship data, allowed for phasing of obtained HLA haplotypes in four of the five individuals. Notably, it has been previously shown that HLA class I alleles can be successfully assigned using OptiType (*50*) in ancient whole-genome shotgun sequencing data (without enrichment) at first-field accuracy (*51*). Here, we demonstrate that combining OptiType, as implemented in the nf-core/HLAtyping pipeline (*49*), with highly resolved pedigrees enables the confident assignment of HLA class I alleles at second-field accuracy as well. This serves as a proof of concept, further supports the reconstructed core pedigree of kindred-1 (Fig. 4), allows for haplotype inference, even in individuals with missing data (mbv111), and provides a practical approach to studying the history and evolution of immunologically relevant variants in the past.

### Imputation, IBD, and hapROH analyses

**Fig. 5.**
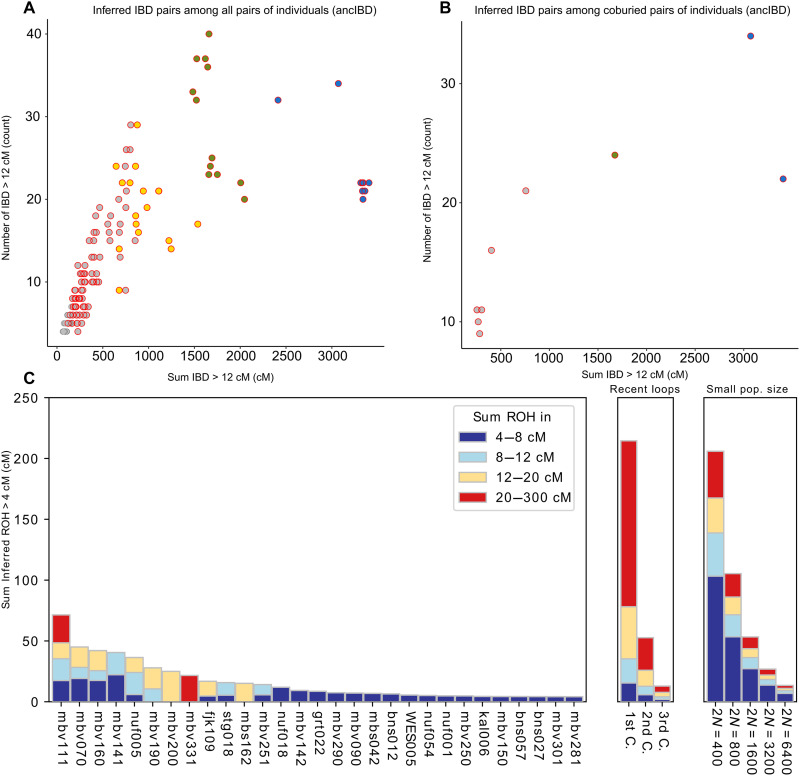
Pairwise IBD sharing and hapROH results. The dots in (A) and (B) represent all pairs of individuals sharing >3 IBD segments >12 cM after application of the default SNP density filter (>220 SNPs/cM) to remove false-positive calls. The points are color coded following Ringbauer *et al.* (*53*) and are defined according to the presumed degree of kinship based on R_ab_ values obtained from ngsRelate analyses with the cutoff values of 0.09375, 0.18375, and 0.375 (i.e., conceptual boundaries between expected values of r for different degrees of kinship: 0, 0.125, 0.25, and 0.5). Red boundaries highlight pairs of individuals sharing ≥3 long IBD segments (>20 cM). (**A**) Results for all pairs of individuals. (**B**) Pairs of individuals interred together. (**C**) HapROH results listing individuals with detected ROH fragments of more than 4 cM and more than 400,000 SNPs from the 1.24 million dataset. The red text highlights individuals from kindred-1.

To further investigate relatedness patterns, we imputed genomes with >0.1× coverage (*n* = 131) using GLIMPSE2 (*52*) and used the resulting genotypes as input for ancIBD (*53*). After filtering [>220 single-nucleotide polymorphisms (SNPs)/cM], 1179 IBD pairs remained (table S10). Pairs sharing ≥3 IBD segments of >12 cM were interpreted as indicative of biological relatedness (Fig. 5A). Although many pairs showed IBD sharing—particularly more distant kinship—few such pairs were detected within multiple burials (Fig. 5B). Kinship categories were inferred using R_ab_ values (*54*), with cutoff values of 0.09375, 0.18375, and 0.375. Red boundaries in Fig. 5 highlight pairs with ≥3 long IBD segments (>20 cM), representing close kinship. The single interments served as a reference for evaluating background IBD sharing within each site, allowing us to contextualize broader patterns of ancestry and relatedness beyond close-kin relationships (figs. S4 and S5).

Last, we analyzed runs of homozygosity (ROHs) to investigate the levels of parental relatedness among tested individuals (*55*) (table S12). All individuals with multiple ROHs ≥ 4 cM were plotted in Fig. 5C. In most of the detected cases, ROHs were short (4 to 8 cM), which is consistent with small ancestral population size and no substantial evidence of recent inbreeding. The few detected exceptions, specifically mbv111 and mbv190, carry ROH sizes suggestive of a certain degree of inbreeding, which could point to their origin in small, isolated communities.

### Background relatedness and population-genetic structure

To assess whether population-genetic structure and background relatedness could influence the observed principal components analysis (PCA) distribution (Fig. 1) and kinship patterns, we evaluated background relatedness using IBD-based network analyses visualizing pairs of individuals sharing at least three segments of ≥8 cM. The visualization revealed pronounced site-specific differences, with substantially higher levels of shared ancestry at Västerhus than at Sigtuna (section S2.5 and figs. S4 and S5). This pervasive background relatedness violates the assumptions underlying many population-genetic summary statistics and can strongly influence PCA clustering and measures of genetic diversity.

We then performed a range of exploratory population genomic analyses, including *f*_3_ and *f*_4_ statistics and multidimensional scaling (MDS) of the resulting distance matrices. Anticipating elevated background relatedness among individuals in this study, we first conducted the *f_3_* and *f_4_* statistics at the individual level after removing related individuals and those with the lowest coverage (tables S13 and S14 and fig. S6). These tests did not reveal statistically robust differentiation between individuals or sites. We additionally computed *f*_3_ statistics incorporating published reference data from Northern Europe spanning the pre-Viking to Early Medieval periods and visualized the results using MDS plots under multiple grouping and subsampling schemes (section S2.5, figs. S7 to S10, and tables S13 and S15), which likewise showed minimal internal structuring, albeit with results pointing to a slight differentiation of Fjälkinge compared to Sigtuna and Västerhus.

Although a reduction in genetic diversity during later Scandinavian periods has been reported previously (*35*), we interpret the apparent reduction in genetic spread observed in our PCA primarily as a consequence of elevated background relatedness at Västerhus, higher heterogeneity in the urban Sigtuna population, and our targeted sampling strategy, rather than a direct signal of broader population-level processes. This interpretation is consistent with earlier work highlighting substantial genetic diversity in medieval urban centers (*35*, *41*).

## DISCUSSION

### Composition of the multiple burials

Almost all multiple burials examined in this study represent primary, simultaneous interments, involving individuals who likely died at or around the same time (*56*), although some cases may reflect sequential multiple burials in which remains were deposited at different times (*56*). A notable pattern is the predominance of same-sex adult-child burials (Fig. 2A). At the same time, individuals interred together rarely exhibited close biological kinship (Figs. 2B, 3, and 5 and table S10). While the absence of close kinship among adults is not unexpected, this finding challenges long-standing assumptions that adults buried with children were typically first-degree relatives.

Previous studies have suggested that some child burials may represent later interments into existing graves (*39*, *40*, *57*) and that coburials of children with adult males may reflect coincidental deaths rather than familial ties (*58*). In our dataset, the overall rarity of close biological kinship within multiple burials likely reflect other forms of social bonds, such as more distant biological relationships, shared origins or backgrounds, or affiliation with a particular household or community. To further explore more distant relatedness, we conducted IBD-based analyses (Fig. 5A), which revealed that although many individuals shared IBD segments consistent with various degrees of kinship, only a small fraction (~6.38%) of these relationships occurred between individuals buried together (Fig. 5B). The presence of diverse degrees of distant relatedness across the cemetery suggests that multiple generations were interred at these sites, without close biological family units being the primary organizing principle of individual multiple burials.

### Gender, age, and spatial patterning

Same-sex adult-child burials lacking close biological relationships (first or second degree) were particularly common in early Christian contexts. Boys and girls were often buried on the same side of the churchyard as adults of the same sex, suggesting that subadults were, in many cases, treated similarly to adults in terms of gendered burial practices. This pattern is especially pronounced at Västerhus but is also observed at other sites, including Sigtuna (mbs021 and mbs022; mbs161 and mbs162) and Lund (win001 and win002).

However, notable exceptions indicate that burial location could sometimes be influenced by social position or contextual factors rather than gender alone. Four infant girls (mbv222, mbv254, mbv260, and mbv311) were buried in the southern part of the churchyard—typically associated with males—and were consistently interred with males, most often adult men or, in one case, young boys. An additional infant girl (mbv341) was buried with males beneath the southern wall of the atrium. Conversely, we did not observe cases of young boys buried with adult women in the northern part of the cemetery. Last, one adult woman (mbv332) was buried within the church, possibly reflecting special status. Thus, mixed-sex burials were more frequent in liminal or symbolically prominent areas, such as along the chancel and beneath the church porch.

### Kinship, kindreds, and social status

To further investigate spatial and social organization, we reconstructed extended pedigrees at Västerhus, where sampling coverage was greatest. These analyses identified apparent connections through extended kindreds, particularly kindred-1, whose members were frequently buried close to the church—suggesting long-standing social positioning or elevated status within the community. One individual associated with this group, wes056 [972 ± 29 BP, Ua-62567]  (*35*), was buried with one of only two scallop shells recovered from Västerhus (Fig. 1C). As Christian burials typically lack grave goods, the presence of the shell—evidence of a completed pilgrimage to Santiago de Compostela (*25*)—is exceptional and points to both mobility and social distinction. Together with her placement within a well-connected kindred, this burial suggests that the woman belonged to the elite and underscores the mobility of selected individuals, particularly women, in Christian traditions.

Pedigree reconstructions further highlight dynamic social processes within the cemetery. At Västerhus, we observe interactions between individuals belonging to founding kindreds and the gradual incorporation of individuals from more distantly related groups. Notably, at least one infant girl (mbv254) interred in a “typically male” burial context was more distantly related to some of the coburied males, underscoring that burial decisions were not solely determined by nuclear family relationships. The high frequency of second-degree relationships across different burials, combined with the rarity of close kinship within individual graves, suggests that concepts of family and fellowship extended well beyond the modern notion of the nuclear household—particularly in rural, close-knit communities such as Västerhus.

### Broader social context and comparative perspectives

In Viking and Early Medieval Scandinavia, households often included extended relatives, servants, employees, and enslaved individuals (*59*). The gradual influence of the church introduced an “ecclesiastical definition of kinship” (*60*), yet social life continued to encompass nonbiological relationships, including fostering, concubinage, apprenticeship, and friendship alliances (*59*, *61*–*65*). Children born out of wedlock could inherit, further complicating the relationship between biological relatedness and social belonging.

The patterns observed in Swedish medieval multiple burials align with a growing body of archaeogenetic research demonstrating that kinship played a central role in community organization across Early Medieval Europe. Continental studies show that burial grounds often structured social space around biological families, with relatives interred in proximity across generations (*66*, *67*). Large-scale genomic analyses further reveal how emerging post-Roman elites organized communities through lineage-based structures, with genetically related individuals occupying prominent burial positions (*68*). Similarly, population-scale genomic studies of the Viking world emphasize kinship networks as fundamental to settlement patterns, mobility, and mortuary practices (*69*).

Our results extend these observations into a later medieval, early Christian Scandinavian context. While kinship and lineage continuity clearly remained important at the community level, multiple burials themselves rarely reflect close biological family units. Instead, these burials appear to have been shaped by households, extended kin groups, or local communities, operating within Christian normative frameworks that may have constrained or overridden purely familial considerations.

### Other factors and limitations

It cannot be ruled out that some multiple burials resulted from pragmatic or coincidental circumstances. Simultaneous deaths due to warfare, accidents, or epidemics could necessitate shared interment; however, the absence of widespread trauma or demographic patterns typical of epidemic mortality (*70*), together with the overall homogeneity of burial contexts, limits our ability to infer specific causes of death.

Our targeted sampling strategy focused on multiple burials and was not designed to capture population-level patterns or enable systematic cross-site comparisons. Instead, it was optimized for investigating biological and social relatedness within burial contexts. Broader inferences will require more comprehensive, site-wide sampling. Nonetheless, the data suggest that early Christian burial norms in Scandinavia—particularly gender-based segregation—were largely followed in practice, including in the burial of children. While boys and girls appear to have been treated similarly in most cases, infants and fetuses were sometimes subject to different burial rules. Considering the identified exceptions, we speculate that some of these individuals may have been unbaptized and therefore ineligible for burial in consecrated ground, illustrating how communities may have flexibly negotiated religious norms to care for their dead.

Although spatial burial placement within churchyards is often interpreted in relation to liturgical or ideological principles, it was likely also shaped by practical considerations, including spatial constraints and the need to position new interments in relation to earlier burials. Burial organization should therefore be understood as the result of intersecting symbolic, social, and pragmatic factors. Preliminary observations further suggest differences between rural (Västerhus) and urban contexts (Sigtuna): Rural communities may have accommodated a wider range of kin relationships within shared burial spaces, reflecting more extended local networks, whereas the more limited urban data indicate a higher prevalence of close biological relationships among individuals buried together. However, these patterns remain tentative and require confirmation through expanded datasets.

Last, we did not perform age-based analyses of spatial burial distribution within the churchyards, as meaningful interpretation would require larger sample sizes. Even at Västerhus, the only fully excavated site, limited numbers constrain robust inference. Preliminary observations nonetheless suggest that burial organization was structured primarily by gender rather than age, with childrenboth under and over 1 year of age distributed in broadly similar ways.

## MATERIALS AND METHODS

### Experimental design

On the basis of available literature, we selected and sampled 118 Late Viking Age to early Medieval individuals deposited in 50 so-called multiple burials from three different burial locations where inhumations containing multiple individuals have been described. Where possible, we selected teeth and petrous bone for DNA extraction (table S2). All destructive sampling for this study was conducted under the appropriate institutional permissions: Newly sampled individuals from Sigtuna were obtained under permits issued by the Sigtuna Museum, and sampling of the Västerhus individuals was approved by the National Historical Museums of Sweden. Individuals from single interments and those from Fjälkinge were previously sampled under permits granted for the ATLAS and 1000 Ancient Genomes projects.

### DNA extraction

DNA extraction and genomic library preparation were conducted in the ancient DNA facility at the Archaeological Research Laboratory within the Department of Archaeology and Classical Studies at Stockholm University. The osteological samples underwent decontamination procedures, including treatment with a 1% bleach solution, rinsing with double-distilled H_2_O, and exposure to ultraviolet light (265 nm, 0.99 J/cm^2^) on both sides. Bone powder was obtained using a DREMEL drill at the lowest setting (5000 rpm). The resulting bone powders underwent overnight lysis at 37°C in 1 ml of extraction buffer comprising 0.5 M EDTA (pH 8.0), 1 M urea, and 2 × 10 μl of proteinase K (10 mg/ml). Subsequently, the lysate underwent concentration using Amicon Ultra (Merck) centrifugal filters and purification through the QIAGEN MinElute Kit (QIAGEN), with a final elution in 2 × 55 μl of EB buffer. The extracted DNA was then stored at −20°C.

### Genomic library preparation and sequencing

Subsequently, 20 μl of the DNA extract was used for genomic library preparation, following the Illumina-compatible protocol established by Meyer and Kircher (*71*). The success of the library construction was assessed through quantitative polymerase chain reaction (PCR) using SYBR Green master mix and primers IS7 and IS8. After confirming the absence of major contamination, the library extraction and library blanks were discontinued at this stage. Meanwhile, the sample libraries underwent PCR amplification split across five parallel 25 μl reactions with indexing primers. The PCR setup included 1× AmpliTaq Gold Buffer, 2.5 mM MgCl_2_, 250 μM of each dNTP, 2.5 U of AmpliTaq Gold (Thermo Fisher Scientific, Waltham, MA), and 200 nM each of the IS4 primer and index primer, following the conditions of 94°C for 10 min, 10 cycles of 94°C for 30 s, 60°C for 30 s, 72°C for 45 s, and a final elongation step of 72°C for 10 min. The amplified libraries were pooled and purified using AMPure XP beads (Agencourt, Beckman Coulter, Brea, CA). Last, the finished libraries’ fragment size and concentration were estimated using a Bioanalyzer with the High Sensitivity Kit (Agilent Technologies, Cary, NC). The libraries were equimolarly pooled and sequenced at the SciLifeLab National Genomics Infrastructure in Stockholm (NGI Stockholm) on the Illumina HiSeqX 10 and/or NovaSeq 6000 platforms. Successful libraries underwent additional rounds of sequencing.

### Sequence postprocessing

Sequences were demultiplexed at NGI Stockholm based on individual index sequences. The resulting FASTQ files underwent trimming and merging processes, requiring a minimum 11-base overlap, performed using either AdapterRemoval v. 2.1.7 (*72*) or Cutadapt v. 2.3 (*73*) and FLASH v. 1.2.11 (*74*). The mapping process used the reference genome build 37 (hg19; hs37d5.fa) with bwa (*75*), using aln parameters: -l 16500 -n 0.01 -o 2. To eliminate PCR duplicates, we used FilterUniqueSAMCons.py (*76*) removing reads shorter than 35 bp or with more than 10% mismatches to the consensus reference sequence. Deamination damage, specifically an excess of transitions at both ends, was examined using misincorp.py. Contamination was assessed through two mitochondrial DNA contamination estimates (*36*, *37*) and an X-chromosome-based contamination analysis incorporated in ANGSD (*38*, *77*). Molecular sex determination was initially accomplished through the Ry-method (*10*) and further verified using the karyo_RxRy_script.py for sex and karyotype detection (*11*).

### Haplogroup assignment

Mitochondrial DNA sequences were extracted from BAM files using SAMtools v. 1.5 (*78*) and then merged into a consensus sequence using a custom Python script (consFastQ2fasta.py). The resulting strict consensus FASTA file was used for mitochondrial DNA haplogroup assignment in HaploGrep 2 (v2.4.0) (*79*).

For Y-chromosome analysis, data were filtered from the BAM files using SAMtools (*78*). Haplogroup-defining SNPs from the minimal reference phylogeny for the human Y chromosome, PhyloTree (*80*), and ISOGG (International Society of Genetic Genealogy, version 15.73) were examined to determine Y haplogroups. To confirm Y-chromosome haplogroup assignments, PathPhynder (*81*) was used with a default mapping quality of 25 and a base quality of 20 in the Best path method. The analysis was limited to transversions.

### Reference panel

To increase the sample size, we combined the newly generated data with 23 relevant genomes from Lund, Sigtuna, and Västerhus, which were published in our earlier studies (*35*, *39*–*41*). The genomic individual data were trimmed by 10 bp from each end and then merged with the Human Origins and 1.24 million Allen Ancient DNA Resource datasets (v50 and v62) (*82*) to compare the individuals with a large panel of modern and ancient samples.

### Statistical analysis

To evaluate the distribution of burial compositions, we used two complementary statistical approaches. First, a Monte Carlo simulation (*n* = 100,000 iterations) was used to determine the probability of specific burial configurations occurring by chance. The null hypothesis assumed an equal probability of any burial being assigned to one of nine possible demographic and sexual composition categories. This method was chosen to assess the significance of the observed frequency of the dominant burial type (same-sex Adult-Child) within the total assemblage (*n* = 51). Second, we used Fisher’s exact test to investigate the association between burial age demographics (Adult-Child, Adult-only, and Child-only) and sexual composition (same-sex versus mixed-sex). All analyses were performed in R (version 4.3.3).

### Kinship analyses

For kinship analyses, we used three different kinship estimation software tools. First, we used READ (*44*). To maximize the number of SNPs for testing, we initially merged our ancient individuals with 10 high-coverage genomes from the Estonian Biocentre Human Genome Diversity Panel (EGDP) (*48*), selecting only transversions. All kinship analyses were performed on the full set of untrimmed individual bam files (*n* = 165).

Second, we estimated the kinship between pairs of individuals using the lcMLkin software (*45*). This software calculates genotype likelihoods instead of relying on observed genotypes, which is advantageous for ancient DNA data due to the tendency of low-coverage genomes to produce false homozygotes. SNPs from the EGDP were selected with a minimum frequency of 0.15, using only transversions to mitigate postmortem damage bias in our samples. Genotype likelihoods for the selected SNPs were called using the script “SNPbam2vcf.py” provided by the authors.

Last, we used the NgsRelate software (*46*, *47*) with default parameters, using a panel of 1,554,712 autosomal transversion SNPs sourced from the EGDP. Population allele frequencies were computed from the whole tested dataset (*n* = 165), encompassing four different sites: Lund (*n* = 2), Sigtuna (*n* = 64), Fjälkinge (*n* = 11), and Västerhus (*n* = 88). To infer pedigree relationships among related individuals, we ran NgsRelate on X-chromosomal loci using a panel of 74,045 transversion SNPs from EGDP. Last, we inferred the most probable relationship and pedigrees by comparing our *k*_0_, *k*_1_, and *k*_2_ values with the expected values for different degrees of relationship as outlined in (*83*) and combining with archaeological, osteological, and uniparental information.

### HLA typing

Medium to high coverage untrimmed human genomes mapped to human reference genome build 37 (hg19) were tested as input for nf-core/HLAtyping pipeline (*49*). To do that we generated more sequencing data from five individuals of known pedigree relation (mbv111, mbv190, mbv250, mbv251, and wes056) obtaining genome coverages ranging between 0.78× and 19× (mean = 12.34×, median = 14.39×) and used nf-core/HLAtyping pipeline to investigate whether it would be possible to assign HLA alleles in ancient DNA in medium coverage ancient genomes in the presence of well-described familial relatedness. The program was able to assign HLA class I alleles (HLA-A, HLA-B, and HLA-C) in all individuals with >10× genome coverage (*n* = 4). Running the analyses on a well-defined ancient kin group we were able to confirm through manual phasing that the obtained alleles could be assigned to defined haplotypes traced through generations and that HLA haplotype of the individual with the most missing data could be inferred (mbv111) (Fig. 4 and fig. S3).

### Imputation, ancIBD, and hapROH analyses

We imputed 131 ancient human genomes with genome coverages of >0.1× using GLIMPSE2 (*52*) and the 1000 Genomes Project reference panel (*84*). The imputed genotypes were subsequently analyzed with ancIBD (*53*) to detect IBD segments shared between pairs of tested individuals. An SNP density filter (>220 SNPs/cM), was applied to filter out false positives. We further used the hapROH software (*55*) to calculate and visualize the ROHs, following the authors’ guidelines (https://pypi.org/project/hapROH/). The IBD networks were generated with Gephi 0.10.1 (*85*) using the Fruchterman Reingold algorithm (*86*).

### Population genomic analyses

PCA was performed using smartpca from the EIGENSOFT package (*87*). A reference panel of West Eurasian populations was selected, and duplicate individuals (e.g., Sanger-sequenced diploid genotypes) were removed. A total of 165 ancient individuals were projected onto a background dataset of 949 present-day individuals using the lsqproj option in smartpca (*87*).

Formal tests of genetic affinity and admixture were conducted by computing *f*_3_ and *f*_4_ statistics using ADMIXTOOLS (*88*), with analyses performed at both the individual and group levels. MDS of *f*_3_-based distance matrices (1 − *f*_3_) was carried out in R (v. 4.4.3) using the cmdscale function for visualization.

## Supplementary Materials

### The PDF file includes:

Supplementary Text Figs. S1 to S10 Table S1 Legends for tables S2 to S15 References

## References

[1] L. Scheuer, S. M. Black, *Developmental Juvenile Osteology* (Academic Press, 2000).

[2] J. E. Buikstra, D. H. Ubelaker, *Standards for Data Collection from Human Skeletal Remains* (Arkansas Archaeological Survey Research Series, 1994).

[3] M. E. Lewis, *The Bioarchaeology of Children: Perspectives from Biological and Forensic Anthropology* (Cambridge Univ. Press, 2009).

[4] T. K. Black III, Sexual dimorphism in the tooth-crown diameters of the deciduous teeth. Am. J. Phys. Anthropol. 48, 77–82 (1978). 623234 10.1002/ajpa.1330480111

[5] D. S. Weaver, Sex differences in the ilia of a known sex and age sample of fetal and infant skeletons. Am. J. Phys. Anthropol. 52, 191–195 (1980). 7369338 10.1002/ajpa.1330520205

[6] H. Schutkowski, Sex determination of infant and juvenile skeletons: I. Morphognostic features. Am. J. Phys. Anthropol. 90, 199–205 (1993). 8430753 10.1002/ajpa.1330900206

[7] A. Veroni, D. Nikitovic, M. A. Schillaci, Brief communication: Sexual dimorphism of the juvenile basicranium. Am. J. Phys. Anthropol. 141, 147–151 (2010). 19937595 10.1002/ajpa.21156

[8] S. Thedéen, Who’s that Girl? The cultural construction of girlhood and the transition to womanhood in Viking Age Gotland. Child. Past 1, 78–93 (2008).

[9] C. Hedenstierna-Jonson, “She came from another place: On the burial of a young girl in Birka (Bj463),” in *Viking Worlds: Things, Spaces and Movement*, M. Hem Erikse, U. Pedersen, B. Runderget, I. Axelsen, H. Lund Berg, Eds. (Oxbow Books, 2014), pp. 90–101.

[10] P. Skoglund, J. Storå, A. Götherström, M. Jakobsson, Accurate sex identification of ancient human remains using DNA shotgun sequencing. J. Archaeol. Sci. 40, 4477–4482 (2013).

[11] K. Anastasiadou, M. Silva, T. Booth, L. Speidel, T. Audsley, C. Barrington, J. Buckberry, D. Fernandes, B. Ford, M. Gibson, A. Gilardet, I. Glocke, K. Keefe, M. Kelly, M. Masters, J. McCabe, L. McIntyre, P. Ponce, S. Rowland, J. Ruiz Ventura, P. Swali, F. Tait, D. Walker, H. Webb, M. Williams, A. Witkin, M. Holst, L. Loe, I. Armit, R. Schulting, P. Skoglund, Detection of chromosomal aneuploidy in ancient genomes. Commun. Biol. 7, 14 (2024). 38212558 10.1038/s42003-023-05642-z PMC10784527

[12] N. A. Stewart, R. F. Gerlach, R. L. Gowland, K. J. Gron, J. Montgomery, Sex determination of human remains from peptides in tooth enamel. Proc. Natl. Acad. Sci. U.S.A. 114, 13649–13654 (2017). 29229823 10.1073/pnas.1714926115 PMC5748210

[13] N. Price, Passing into poetry: Viking-Age mortuary drama and the origins of norse mythology. Mediev. Archaeol. 54, 123–156 (2010).

[14] A. Hed Jakobsson, J. Runer, A. Kjellström, T. Björk, *I Sigtunas utkant. Slutundersökningsrapport över gravar och bebyggelse vid Götes Mack* (Arkeologikonsult, 2017).

[15] E. Naumann, M. Krzewińska, A. Götherström, G. Eriksson, Slaves as burial gifts in Viking Age Norway? Evidence from stable isotope and ancient DNA analyses. J. Archaeol. Sci. 41, 533–540 (2014).

[16] A. Kjellström, “The norm and the subaltern: Identifying slaves in an early medieval Scandinavian society,” in *The Archaeology of Slavery in Early Medieval Northern Europe: The Invisible Commodity*, F. Biermann, M. Jankowiak, Eds. (Springer International Publishing, 2021), pp. 67–79.

[17] A. Pedersen, “Grave, høje og kirker- gravskikke og monumenter mellom hedensk og kristet i Danmark,” in *Från Hedniskt till Kristet. Förändringar i Begravningsbruk Och Gravskick i Skandinavien c:A 800–1200*, B. Nilsson, Ed. (Sällskapet Runica et Mediaevalia, 2010), pp. 57–102.

[18] A.-S. Gräslunde, “Religionsskiftet speglat i gravskicket.: Ny svensk forskning kring senvikingatida gravar och gravskick,” in *Från Hedniskt till Kristet. Förändringar i Begravningsbruk Och Gravskick i Skandinavien c:A 800–1200*, B. Nilsson, Ed. (Sällskapet Runica et Mediaevalia, 2010), pp. 131–164.

[19] B. Nilsson, “Den tidiga medeltidens likbegängelse och begravningsbruk i Västkyrkan,” in *Från Hedniskt till Kristet. Förändringar i Begravningsbruk Och Gravskick i Skandinavien c:A 800–1200*, B. Nilsson, Ed. (Sällskapet Runica et Mediaevalia, 2010), pp. 9–56.

[20] B. Nilsson, *Kvinnor, Män Och Barn På Medeltida Begravningsplatser: Women, Men and Children in Mediaeval Burial Places* (Lunne böcker, 1994).

[21] S. Nordeide, ““I døden er vi alle ulike”: Gravmateriale i Sør-Norge i yngre jernalder og tidlig middelalder som grunnlag for å belyse kristningen av Norge,” in *Från Hedniskt till Kristet: Förändringar i Begravningsbruk Och Gravskick i Skandinavien ca. 800–1200*, B. Nilsson, Ed. (Sällskapet Runica et Mediavalia, 2010), pp. 102–130.

[22] R. Gilchrist, “Transforming medieval beliefs. The significance of bodily resurrection to medieval burial rituals,” in *Death and Changing Rituals. Function and Meaning in Ancient Funerary Practices*, J. R. Brandt, H. Ingvaldsen, M. Prusac, Eds. (Oxbow Books, 2014), pp. 379–396.

[23] A. Andrén, Ad sanctos – De dödas plats under medeltiden. Hikuin 27, 7 (2000).

[24] K. Jonsson, “Practices for the living and the dead: Medieval and post-Reformation burials in Scandinavia,” thesis, Stockholm University, Stockholm (2009).

[25] N. G. Gejvall, *Westerhus: Medieval Population and Church in the Light of Skeletal Remains* (Kungl. Vitterhets-, historie- och antikvitetsakademien, 1960) Monografier 43.

[26] J. Kieffer-Olsen, “Grav og gravskik i det middelalderlige Danmark: 8 kirkegårdsudgravninger,” thesis, Aarhus Universitet, Højbjerg (1993).

[27] K. Jonsson, “Till döden skiljer oss åt…,” in *Västerhus: Kapell, kyrkogård och befolkning*, E. Iregren, V. Alexandersen, L. Redin, Eds. (Kungliga Vitterhets- historie- och antikvitetsakademien, 2009), pp. 40–63.

[28] B. Nilsson, “Död och begravning. Begravningsskicket i Norden,” in *Tanke Och Tro: Aspekter På Medeltidens Tankevärld Och Fromhetsliv*, vol. 3 of *Studier till det medeltida Sverige*, O. Ferm, I. Estham, G. Tegnér, Eds. (Riksantikvarieämbetet, 1987), pp. 133–150.

[29] B. Nilsson, “De sepulturis. Gravrätten i Corpus Iuris Canonici och medeltida nordisk lagstiftning,” thesis, Stockholm University, Stockholm (1989).

[30] M. Ranåker, “Flerpersonsgravar under medeltid. Västerhus kyrkogård belyst av andra begravningsplatser,” in *Västerhus. Kapell, kyrkogård och befolkning.*, E. Iregren, V. Alexandersen, L. Redin, Eds. (Kungliga Vitterhets- historie- och antikvitetsakademien, 2009), pp. 26–39.

[31] K. W. Alt, W. Vach, “Kinship studies in skeletal remains - Concepts and examples,” in *Dental Anthropology: Fundamentals, Limits and Prospects*, K. W. Alt, F. W. Rösing, M. Teschler-Nicola, Eds. (Springer Vienna, 1998), pp. 537–554.

[32] C. F. Ratican, *Bodies, Beings, and the Multiple Burial Rite of the Western Viking World* (Routledge, 2024).

[33] K. M. Johnson, K. S. Paul, Bioarchaeology and kinship: Integrating theory, social relatedness, and biology in ancient family research. J. Archaeol. Res. 24, 75–123 (2015).

[34] A. Kjellström, “Tillsammans i evighet: Osteologiska aspekter på flerpersonsgravar i Sigtuna,” in *Sigtuna Dei* (Sigtuna museer, 2010), pp. 127–136.

[35] R. Rodríguez-Varela, K. H. S. Moore, S. S. Ebenesersdóttir, G. M. Kilinc, A. Kjellström, L. Papmehl-Dufay, C. Alfsdotter, B. Berglund, L. Alrawi, N. Kashuba, V. Sobrado, V. K. Lagerholm, E. Gilbert, G. L. Cavalleri, E. Hovig, I. Kockum, T. Olsson, L. Alfredsson, T. F. Hansen, T. Werge, A. R. Munters, C. Bernhardsson, B. Skar, A. Christophersen, G. Turner-Walker, S. Gopalakrishnan, E. Daskalaki, A. Omrak, P. Pérez-Ramallo, P. Skoglund, L. Girdland-Flink, F. Gunnarsson, C. Hedenstierna-Jonson, M. T. P. Gilbert, K. Lidén, M. Jakobsson, L. Einarsson, H. Victor, M. Krzewińska, T. Zachrisson, J. Storå, K. Stefánsson, A. Helgason, A. Götherström, The genetic history of Scandinavia from the Roman Iron Age to the present. Cell 186, 32–46.e19 (2023). 36608656 10.1016/j.cell.2022.11.024

[36] R. E. Green, A. W. Briggs, J. Krause, K. Prüfer, H. A. Burbano, M. Siebauer, M. Lachmann, S. Pääbo, The Neandertal genome and ancient DNA authenticity. EMBO J. 28, 2494–2502 (2009). 19661919 10.1038/emboj.2009.222 PMC2725275

[37] Q. Fu, H. Li, P. Moorjani, F. Jay, S. M. Slepchenko, A. A. Bondarev, P. L. F. Johnson, A. Aximu-Petri, K. Prüfer, C. de Filippo, M. Meyer, N. Zwyns, D. C. Salazar-García, Y. V. Kuzmin, S. G. Keates, P. A. Kosintsev, D. I. Razhev, M. P. Richards, N. V. Peristov, M. Lachmann, K. Douka, T. F. G. Higham, M. Slatkin, J.-J. Hublin, D. Reich, J. Kelso, T. B. Viola, S. Pääbo, Genome sequence of a 45,000-year-old modern human from western Siberia. Nature 514, 445–449 (2014). 25341783 10.1038/nature13810 PMC4753769

[38] M. Rasmussen, X. Guo, Y. Wang, K. E. Lohmueller, S. Rasmussen, A. Albrechtsen, L. Skotte, S. Lindgreen, M. Metspalu, T. Jombart, T. Kivisild, W. Zhai, A. Eriksson, A. Manica, L. Orlando, F. M. De La Vega, S. Tridico, E. Metspalu, K. Nielsen, M. C. Ávila-Arcos, J. V. Moreno-Mayar, C. Muller, J. Dortch, M. T. P. Gilbert, O. Lund, A. Wesolowska, M. Karmin, L. A. Weinert, B. Wang, J. Li, S. Tai, F. Xiao, T. Hanihara, G. van Driem, A. R. Jha, F.-X. Ricaut, P. de Knijff, A. B. Migliano, I. G. Romero, K. Kristiansen, D. M. Lambert, S. Brunak, P. Forster, B. Brinkmann, O. Nehlich, M. Bunce, M. Richards, R. Gupta, C. D. Bustamante, A. Krogh, R. A. Foley, M. M. Lahr, F. Balloux, T. Sicheritz-Pontén, R. Villems, R. Nielsen, J. Wang, E. Willerslev, An aboriginal Australian genome reveals separate human dispersals into Asia. Science 334, 94–98 (2011). 21940856 10.1126/science.1211177 PMC3991479

[39] M. Krzewińska, R. Rodríguez-Varela, C. Ahlström Arcini, T. Ahlström, N. Hertzman, J. Storå, A. Götherström, Related in death? A curious case of a foetus hidden in bishop Peder Winstrup’s coffin in Lund, Sweden. J. Archaeol. Sci. Rep. 37, 102939 (2021).

[40] M. Krzewińska, R. Rodríguez-Varela, R. Yaka, M. Vicente, G. Runfeldt, M. Sager, C. Ahlström Arcini, T. Ahlström, N. Hertzman, J. Storå, A. Götherström, Related in death? Further insights on the curious case of Bishop Peder Winstrup and his grandchild’s burial. Heritage 7, 576–584 (2024).

[41] M. Krzewińska, A. Kjellström, T. Günther, C. Hedenstierna-Jonson, T. Zachrisson, A. Omrak, R. Yaka, G. M. Kılınç, M. Somel, V. Sobrado, J. Evans, C. Knipper, M. Jakobsson, J. Storå, A. Götherström, Genomic and strontium isotope variation reveal immigration patterns in a Viking Age Town. Curr. Biol. 28, 2730–2738.e10 (2018). 30146150 10.1016/j.cub.2018.06.053

[42] A. Kjellström, *The Urban Farmer: Osteoarchaeological Analysis of Skeletons from Medieval Sigtuna Interpreted in a Socioeconomic Perspective* (Osteoarchaeological Research Laboratory, Stockholm University, 2005), theses and papers in Osteoarchaeology 2.

[43] L. Mejsholm, “Constructions of early childhood at the syncretic cemetery of Fjälkinge: A case study,” in *Youth and Age in the Medieval North* (Brill, 2008), pp. 37–56.

[44] J. M. Monroy Kuhn, M. Jakobsson, T. Günther, Estimating genetic kin relationships in prehistoric populations. PLOS ONE 13, e0195491 (2018). 29684051 10.1371/journal.pone.0195491 PMC5912749

[45] M. Lipatov, K. Sanjeev, R. Patro, K. Veeramah, Maximum likelihood estimation of biological relatedness from low coverage sequencing data. bioRxiv 023374 [Preprint] (2015). 10.1101/023374.

[46] K. Hanghøj, I. Moltke, P. A. Andersen, A. Manica, T. S. Korneliussen, Fast and accurate relatedness estimation from high-throughput sequencing data in the presence of inbreeding. GigaScience 8, giz034 (2019). 31042285 10.1093/gigascience/giz034 PMC6488770

[47] T. S. Korneliussen, I. Moltke, NgsRelate: A software tool for estimating pairwise relatedness from next-generation sequencing data. Bioinformatics 31, 4009–4011 (2015). 26323718 10.1093/bioinformatics/btv509 PMC4673978

[48] L. Pagani, D. J. Lawson, E. Jagoda, A. Mörseburg, A. Eriksson, M. Mitt, F. Clemente, G. Hudjashov, M. DeGiorgio, L. Saag, J. D. Wall, A. Cardona, R. Mägi, M. A. W. Sayres, S. Kaewert, C. Inchley, C. L. Scheib, M. Järve, M. Karmin, G. S. Jacobs, T. Antao, F. M. Iliescu, A. Kushniarevich, Q. Ayub, C. Tyler-Smith, Y. Xue, B. Yunusbayev, K. Tambets, C. B. Mallick, L. Saag, E. Pocheshkhova, G. Andriadze, C. Muller, M. C. Westaway, D. M. Lambert, G. Zoraqi, S. Turdikulova, D. Dalimova, Z. Sabitov, G. N. N. Sultana, J. Lachance, S. Tishkoff, K. Momynaliev, J. Isakova, L. D. Damba, M. Gubina, P. Nymadawa, I. Evseeva, L. Atramentova, O. Utevska, F.-X. Ricaut, N. Brucato, H. Sudoyo, T. Letellier, M. P. Cox, N. A. Barashkov, V. Škaro, L. Mulahasanovic, D. Primorac, H. Sahakyan, M. Mormina, C. A. Eichstaedt, D. V. Lichman, S. Abdullah, G. Chaubey, J. T. S. Wee, E. Mihailov, A. Karunas, S. Litvinov, R. Khusainova, N. Ekomasova, V. Akhmetova, I. Khidiyatova, D. Marjanović, L. Yepiskoposyan, D. M. Behar, E. Balanovska, A. Metspalu, M. Derenko, B. Malyarchuk, M. Voevoda, S. A. Fedorova, L. P. Osipova, M. M. Lahr, P. Gerbault, M. Leavesley, A. B. Migliano, M. Petraglia, O. Balanovsky, E. K. Khusnutdinova, E. Metspalu, M. G. Thomas, A. Manica, R. Nielsen, R. Villems, E. Willerslev, T. Kivisild, M. Metspalu, Genomic analyses inform on migration events during the peopling of Eurasia. Nature 538, 238–242 (2016). 27654910 10.1038/nature19792 PMC5164938

[49] P. A. Ewels, A. Peltzer, S. Fillinger, H. Patel, J. Alneberg, A. Wilm, M. U. Garcia, P. Di Tommaso, S. Nahnsen, The nf-core framework for community-curated bioinformatics pipelines. Nat. Biotechnol. 38, 276–278 (2020). 32055031 10.1038/s41587-020-0439-x

[50] A. Szolek, B. Schubert, C. Mohr, M. Sturm, M. Feldhahn, O. Kohlbacher, OptiType: Precision HLA typing from next-generation sequencing data. Bioinformatics 30, 3310–3316 (2014). 25143287 10.1093/bioinformatics/btu548 PMC4441069

[51] A. G. Plascencia, M. Jakobsson, F. Sánchez-Quinto, Ancient DNA HLA typing reveals significant shifts in frequency in Europe since the Neolithic. Sci. Rep. 15, 6161 (2025). 39979344 10.1038/s41598-024-82449-w PMC11842861

[52] S. Rubinacci, D. M. Ribeiro, R. J. Hofmeister, O. Delaneau, Efficient phasing and imputation of low-coverage sequencing data using large reference panels. Nat. Genet. 53, 120–126 (2021). 33414550 10.1038/s41588-020-00756-0

[53] H. Ringbauer, Y. Huang, A. Akbari, S. Mallick, I. Olalde, N. Patterson, D. Reich, Accurate detection of identity-by-descent segments in human ancient DNA. Nat. Genet. 56, 143–151 (2023). 38123640 10.1038/s41588-023-01582-w PMC10786714

[54] P. W. Hedrick, R. C. Lacy, Measuring relatedness between inbred individuals. J. Hered. 106, 20–25 (2015). 25472983 10.1093/jhered/esu072

[55] H. Ringbauer, J. Novembre, M. Steinrücken, Parental relatedness through time revealed by runs of homozygosity in ancient DNA. Nat. Commun. 12, 5425 (2021). 34521843 10.1038/s41467-021-25289-w PMC8440622

[56] G. Miniaci, Multiple burials in ancient societies: Theory and methods from Egyptian archaeology. Camb. Archaeol. J. 29, 287–307 (2019).

[57] S. Crawford, G. Shepherd, “Children, childhood and society,” in *IAA Interdisciplinary Series Vol. I: Studies in Archaeology, History, Literature and Art*, S. Crawford, G. Shepherd, Eds. (BAR International Series 1696, 2007), pp. 1–4.

[58] C. A. Roberts, M. Cox, *Health & Disease in Britain: From Prehistory to the Present Day* (Sutton Publishing, 2003).

[59] J. V. Sigurðsson, *Viking Friendship: The Social Bond in Iceland and Norway, c. 900–1300* (Cornell Univ. Press, 2017).

[60] H. Vogt, *The Function of Kinship in Medieval Nordic Legislation* (Brill, ed. 1, 2010).

[61] A. Magnúsdóttir, “Frillor och fruar. Politik och samlevnad på Island 1120–1400,” thesis, University of Gothenburg, Gothenburg (2001).

[62] A. G. Magnúsdóttir, “Women and sexual politics,” in *The Viking World*, S. Brink, N. Price, Eds. (Routledge, 2008), pp. 40–48.

[63] R. M. Karras, Concubinage and slavery in the Viking Age. Scand. Stud. 62, 141–162 (1990).

[64] T. Nors, Illegitimate children and their high-born mothers. Scand. J. Hist. 21, 17–37 (1996).

[65] A. Hansen, “Fosterage and dependency in medieval Iceland and its significance in Gísla Saga,” in *Youth and Age in Medieval North*, S. Lewis-Simpson, Ed. (Brill, 2008), pp. 73–86.

[66] C. E. G. Amorim, S. Vai, C. Posth, A. Modi, I. Koncz, S. Hakenbeck, M. C. La Rocca, B. Mende, D. Bobo, W. Pohl, L. P. Baricco, E. Bedini, P. Francalacci, C. Giostra, T. Vida, D. Winger, U. von Freeden, S. Ghirotto, M. Lari, G. Barbujani, J. Krause, D. Caramelli, P. J. Geary, K. R. Veeramah, Understanding 6th-century barbarian social organization and migration through paleogenomics. Nat. Commun. 9, 3547 (2018). 30206220 10.1038/s41467-018-06024-4 PMC6134036

[67] K. Wang, B. Tobias, D. Pany-Kucera, M. Berner, S. Eggers, G. A. Gnecchi-Ruscone, D. Zlámalová, J. Gretzinger, P. Ingrová, A. B. Rohrlach, J. Tuke, L. Traverso, P. Klostermann, R. Koger, R. Friedrich, K. Wiltschke-Schrotta, S. Kirchengast, S. Liccardo, S. Wabnitz, T. Vida, P. J. Geary, F. Daim, W. Pohl, J. Krause, Z. Hofmanová, Ancient DNA reveals reproductive barrier despite shared Avar-period culture. Nature 638, 1007–1014 (2025). 39814885 10.1038/s41586-024-08418-5 PMC11864967

[68] Y. Tian, I. Koncz, S. Defant, C. Giostra, D. N. Vyas, A. Sołtysiak, L. Pejrani Baricco, R. Fetner, C. Posth, G. Brandt, E. Bedini, A. Modi, M. Lari, S. Vai, P. Francalacci, R. Fernandes, A. Steinhof, W. Pohl, D. Caramelli, J. Krause, A. Izdebski, P. J. Geary, K. R. Veeramah, The role of emerging elites in the formation and development of communities after the fall of the Roman Empire. Proc. Natl. Acad. Sci. U.S.A. 121, e2317868121 (2024). 39159385 10.1073/pnas.2317868121 PMC11388374

[69] A. Margaryan, D. J. Lawson, M. Sikora, F. Racimo, S. Rasmussen, I. Moltke, L. M. Cassidy, E. Jørsboe, A. Ingason, M. W. Pedersen, T. Korneliussen, H. Wilhelmson, M. M. Buś, P. de Barros Damgaard, R. Martiniano, G. Renaud, C. Bhérer, J. V. Moreno-Mayar, A. K. Fotakis, M. Allen, R. Allmäe, M. Molak, E. Cappellini, G. Scorrano, H. McColl, A. Buzhilova, A. Fox, A. Albrechtsen, B. Schütz, B. Skar, C. Arcini, C. Falys, C. H. Jonson, D. Błaszczyk, D. Pezhemsky, G. Turner-Walker, H. Gestsdóttir, I. Lundstrøm, I. Gustin, I. Mainland, I. Potekhina, I. M. Muntoni, J. Cheng, J. Stenderup, J. Ma, J. Gibson, J. Peets, J. Gustafsson, K. H. Iversen, L. Simpson, L. Strand, L. Loe, M. Sikora, M. Florek, M. Vretemark, M. Redknap, M. Bajka, T. Pushkina, M. Søvsø, N. Grigoreva, T. Christensen, O. Kastholm, O. Uldum, P. Favia, P. Holck, S. Sten, S. V. Arge, S. Ellingvåg, V. Moiseyev, W. Bogdanowicz, Y. Magnusson, L. Orlando, P. Pentz, M. D. Jessen, A. Pedersen, M. Collard, D. G. Bradley, M. L. Jørkov, J. Arneborg, N. Lynnerup, N. Price, M. T. P. Gilbert, M. E. Allentoft, J. Bill, S. M. Sindbæk, L. Hedeager, K. Kristiansen, R. Nielsen, T. Werge, E. Willerslev, Population genomics of the Viking world. Nature 585, 390–396 (2020). 32939067 10.1038/s41586-020-2688-8

[70] D. Castex, S. Kacki, Demographic patterns distinctive of epidemic cemeteries in archaeological samples. Microbiol. Spectr. 4, 10.1128/microbiolspec.PoH-0015-2015 (2016). 10.1128/microbiolspec.PoH-0015-2015 27726822

[71] M. Meyer, M. Kircher, Illumina sequencing library preparation for highly multiplexed target capture and sequencing. Cold Spring Harb. Protoc. 6, pdb.prot5448 (2010). 10.1101/pdb.prot5448 20516186

[72] M. Schubert, S. Lindgreen, L. Orlando, AdapterRemoval v2: Rapid adapter trimming, identification, and read merging. BMC. Res. Notes 9, 88 (2016). 26868221 10.1186/s13104-016-1900-2 PMC4751634

[73] M. Martin, Cutadapt removes adapter sequences from high-throughput sequencing reads. EMBnet. J. 17, 10.14806/ej.17.1.200 (2011).

[74] T. Magoč, S. L. Salzberg, FLASH: Fast length adjustment of short reads to improve genome assemblies. Bioinformatics 27, 2957–2963 (2011). 21903629 10.1093/bioinformatics/btr507 PMC3198573

[75] H. Li, R. Durbin, Fast and accurate long-read alignment with Burrows–Wheeler transform. Bioinformatics 26, 589–595 (2010). 20080505 10.1093/bioinformatics/btp698 PMC2828108

[76] M. Kircher, “Analysis of high-throughput Ancient DNA sequencing data,” in *Ancient DNA*, vol. 840 of *Methods in Molecular Biology*, B. Shapiro, M. Hofreiter, Eds. (Humana Press, 2012), pp. 197–228. 10.1007/978-1-61779-516-9_23 22237537

[77] T. S. Korneliussen, A. Albrechtsen, R. Nielsen, ANGSD: Analysis of next generation sequencing data. BMC Bioinformatics 15, 356 (2014). 25420514 10.1186/s12859-014-0356-4 PMC4248462

[78] H. Li, B. Handsaker, A. Wysoker, T. Fennell, J. Ruan, N. Homer, G. Marth, G. Abecasis, R. Durbin, 1000 Genomes Project Data Processing Subgroup, The sequence alignment/map format and SAMtools. Bioinformatics 25, 2078–2079 (2009). 19505943 10.1093/bioinformatics/btp352 PMC2723002

[79] H. Weissensteiner, D. Pacher, A. Kloss-Brandstätter, L. Forer, G. Specht, H.-J. Bandelt, F. Kronenberg, A. Salas, S. Schönherr, HaploGrep 2: Mitochondrial haplogroup classification in the era of high-throughput sequencing. Nucleic Acids Res. 44, W58–W63 (2016). 27084951 10.1093/nar/gkw233 PMC4987869

[80] M. van Oven, A. Van Geystelen, M. Kayser, R. Decorte, M. H. D. Larmuseau, Seeing the wood for the trees: A minimal reference phylogeny for the human Y chromosome. Hum. Mutat. 35, 187–191 (2014). 24166809 10.1002/humu.22468

[81] R. Martiniano, B. De Sanctis, P. Hallast, R. Durbin, Placing ancient DNA sequences into reference phylogenies. Mol. Biol. Evol. 39, msac017 (2022). 35084493 10.1093/molbev/msac017 PMC8857924

[82] S. Mallick, A. Micco, M. Mah, H. Ringbauer, I. Lazaridis, I. Olalde, N. Patterson, D. Reich, The Allen Ancient DNA Resource (AADR) a curated compendium of ancient human genomes. Sci. Data 11, 182 (2024). 38341426 10.1038/s41597-024-03031-7 PMC10858950

[83] B. S. Weir, A. D. Anderson, A. B. Hepler, Genetic relatedness analysis: Modern data and new challenges. Nat. Rev. Genet. 7, 771–780 (2006). 16983373 10.1038/nrg1960

[84] 1000 Genomes Project Consortium, G. R. Abecasis, A. Auton, L. D. Brooks, M. A. De Pristo, R. M. Durbin, R. E. Handsaker, H. M. Kang, G. T. Marth, G. A. McVean, An integrated map of genetic variation from 1,092 human genomes. Nature 491, 56–65 (2012). 23128226 10.1038/nature11632 PMC3498066

[85] M. Bastian, S. Heymann, M. Jacomy, Gephi: An open source software for exploring and manipulating networks. Proc. Int. AAAI Conf. Web Soc. Media 3, 361–362 (2009).

[86] T. M. J. Fruchterman, E. M. Reingold, Graph drawing by force-directed placement. Softw Pract Exp 21, 1129–1164 (1991).

[87] N. Patterson, A. L. Price, D. Reich, Population structure and eigenanalysis. PLOS Genet. 2, e190 (2006). 17194218 10.1371/journal.pgen.0020190 PMC1713260

[88] N. Patterson, P. Moorjani, Y. Luo, S. Mallick, N. Rohland, Y. Zhan, T. Genschoreck, T. Webster, D. Reich, Ancient admixture in human history. Genetics 192, 1065–1093 (2012). 22960212 10.1534/genetics.112.145037 PMC3522152

[89] F. L. Adair, R. E. Scammon, A study of the ossification centers of the wrist, knee and ankle at birth, with particular reference to the physical development and maturity of the newborn. Am. J. Obstet. Gynecol. 2, 35–60 (1921).

[90] L. Scheuer, S. M. Black, *The Juvenile Skeleton* (Elsevier Science, 2004).

[91] J. Bruzek, A method for visual determination of sex, using the human hip bone. Am. J. Phys. Anthropol. 117, 157–168 (2002). 11815949 10.1002/ajpa.10012

[92] V. Novotný, Sex determination of the pelvic bone: A systems approach. Anthropologie 24, 197–206 (1986).

[93] T. W. Phenice, A newly developed visual method of sexing the os pubis. Am. J. Phys. Anthropol. 30, 297–301 (1969). 5772048 10.1002/ajpa.1330300214

[94] A. Kemkes-Grottenthaler, F. Löbig, F. Stock, Mandibular ramus flexure and gonial eversion as morphologic indicators of sex. Homo 53, 97–111 (2002). 12489410 10.1078/0018-442x-00039

[95] P. L. Walker, Sexing skulls using discriminant function analysis of visually assessed traits. Am. J. Phys. Anthropol. 136, 39–50 (2008). 18324631 10.1002/ajpa.20776

[96] D. R. Hunt, Sex determination in the subadult ilia: An indirect test of Weaver’s nonmetric sexing method. J. Forensic Sci. 35, 881–885 (1990). 2391479

[97] J. Irurita Olivares, I. Alemán Aguilera, Validation of the sex estimation method elaborated by Schutkowski in the Granada Osteological Collection of identified infant and young children: Analysis of the controversy between the different ways of analyzing and interpreting the results. Int. J. Leg. Med. 130, 1623–1632 (2016). 10.1007/s00414-016-1354-z 27002628

[98] D. M. Mittler, S. G. Sheridan, Sex determination in subadults using auricular surface morphology: A forensic science perspective. J. Forensic Sci. 37, 1068–1075 (1992). 1506828

[99] L. Scheuer, A blind test of mandibular morphology for sexing mandibles in the first few years of life. Am. J. Phys. Anthropol. 119, 189–191 (2002). 12237939 10.1002/ajpa.10098

[100] R. C. Sutter, Nonmetric subadult skeletal sexing traits: I. A blind test of the accuracy of eight previously proposed methods using prehistoric known-sex mummies from northern Chile. J. Forensic Sci. 48, 927–935 (2003). 14535657

[101] S. Thedéen, “Immortal maidens: The visual significance of the colour white in girls’ graves on Viking-Age Gotland,” in *Making Sense of Things: Archaeologies of Sensory Perception. Stockholm Studies in Archaeology* (Department of Archaeology and Classical Studies, Stockholm University, 2010), vol. 53, pp. 103–120.

[102] M. L. Stig Sørensen, *Gender Archaeology* (Polity Press, 2000).

[103] M. Ivarsson-Aalders, M. Krzewińska, E. Karlsson, A. Götherström, A. Kjellström , Beyond the binary? A multi-method approach to sexing children at the Viking Age Site of Ihre, Gotland. Int. J. Osteoarchaeol. 35, 233–247 (2025).

[104] H. Guy, C. Masset, C.-A. Baud, Infant taphonomy. Int. J. Osteoarchaeol. 7, 221–229 (1997).

[105] S. E. Halcrow, N. Tayles, “The bioarchaeological investigation of children and childhood,” in *Social Bioarchaeology*, vol 14 of *Blackwell Studies in Global Archaeology*, S. C. Agarwal, B. A. Glencross, Eds. (Blackwell Publishing Ltd., 2011), pp. 333–360.

[106] S. Crawford, C. Lewis, Childhood studies and the society for the study of childhood in the past. Child. Past 1, 5–16 (2008).

[107] D. Pany-Kucera, K. Rebay-Salisbury, *Ages and Abilities: The Stages of Childhood and Their Social Recognition in Prehistoric Europe and Beyond* (Archaeopress, 2020).

[108] C. E. Batey, C. Paterson, “A Viking burial at Balnakeil, Sutherland,” in *Early Medieval Art and Archaeology in the Northern World: Studies in Honour of James Graham-Campbell*, vol. 58 of Series: Northern World, A. Reynolds, L. Webster, Eds. (Brill, 2012), pp. 631–659.

[109] C. Callow, “Transitions to Adulthood in Early Icelandic Society” in *Children, Childhood and Social Identity*, IAA Interdisciplinary Series, vol. 1 of *Studies in Archaeology, History, Literature and Art*, S. Crawford, G. Shepherd, Eds. (BAR International Series 1696, 2007), pp. 45–55.

[110] P. G. Foote, D. M. Wilson, *The Viking Achievement: The Society and Culture of Early Medieval Scandinavia* (Sidgwick & Jackson, 1970).

[111] J. Myrdal, G. Bäärnhielm, *Kvinnor, Barn & Fester i Medeltida Mirakelberättelser: Med En Katalog Över Svenska Mirakelberättelser Och En Nyöversättning Av Brynolfsmiraklerna* (Skaraborgs länsmuseum, 1994).

[112] E. Iregren, Scandinavian women during the medieval period; health, childbirth and childcare. Collegium Anthropologicum 16, 59–81 (1992).

[113] N. Price, “Dying and the dead: Viking Age mortuary behaviour,” in *The Viking World* (Routledge, 2008), pp. 257–273.

[114] A. M. Hållans Stenholm, *Fornminnen: Det Förflutnas Roll i Det Förkristna Och Kristna Mälardalen* (Nordic Academic Press, 2012), vol. 15.

[115] I. Barbiera, Buried together, buried alone: Christian commemoration and kinship in the early Middle Ages. Early Mediev. Eur. 23, 385–409 (2015).

[116] P. Holck, *Cremated Bones: A Medical-Anthropological Study of an Archaeological Material on Cremation Burials* (Universitetet i Oslo, 1996).

[117] C. Ekholst, “För varje brottsling ett straff: Föreställningar om kön i de svenska medeltidslagarna,” thesis, Historiska Institutionen, Stockholm (2009).

[118] A. Ney, *Vänskap Mellan Kvinnor På Vikingatiden: Om Urval Och Historieskrivning i de Isländska Sagorna* (Nordic Academic Press, 2024).

[119] E. Iregren, V. Alexandersen, H. Jungner, P. Isberg, “Diet and growth of medieval children in a rural society in Northern Europe,” in *Dieta y Crecimiento Infantil Medieval en una Sociedad Rural del Norte de Europa*, P. Montero, C. Prado, P. Acevedo Cantero, M. Carmenate, A. del Valle, J. Herrerín, J. F. Romero, K. Keller, N. López, A. I. Mora, Eds. (Sociedad Española de Antropología Física, 2015), pp. 391–406.

[120] T. Zachrisson, C. Ljung, A. Kjellström, “Skärningspunkt Sigtuna: En första presentation av ett forskningsprojekt,” *Situne Dei: Årsskrift för Sigtunaforskning och historisk arkeologi* (Sigtuna Museum, 2017), pp. 52–63.

[121] R. Edberg, “No kingdom without a town. Anund Olofsson’s policy for national independence and its materiality,” in *New Aspects on Viking-Age Urbanism c. AD 750–1100. Proceedings of the International Symposium at the Swedish History Museum, April 17th–20th 2013.*, L. Holmquist, S. Kalmring, C. Hedenstierna-Jonson, Eds. (Archaeological Research Laboratory, 2016), pp. 151–158.

[122] J. Ros, “Sigtuna and the excavations at the Urmakaren and Trädgårdsmästaren sites,” in *New Aspects on Viking-Age Urbanism c. AD 750–1100: Proceedings of the International Symposium at the Swedish History Museum, April 17-20th 2013.*, L. Holmquist, S. Kalmring, C. Hedenstierna-Jonson, Eds. (Archaeological Research Laboratory, 2016), pp. 139–150.

[123] J. Ros, *Sigtuna: The Town, the Churches and the Ecclesiastical Organisation*, vol. 30 of *Occasional Papers in Archaeology* (Uppsala Univ., 2001), 1100–6358.

[124] S. Tesch, “Skilda gravar, skilda världar - tidigkristna gravar, kyrkor, stadsgårdar och storgårdar i Sigtuna och Mälarområdet,” in *Medeltida Storgårdar: 15 Uppsatser Om Ett Tvärvetenskapligt Forskningsproblem* (Gustav Adolfs Akademien, 2014), pp. 101–130.

[125] M. Roslund, “Bridging two worlds: Tracing merchants from the Holy Roman Empire in high medieval Sigtuna,” in *Zwischen Fjorden Und Steppe: Festschrift Für Johan Callmer Zum 65. Geburtstag*, C. Theune, F. Biermann, R. Struwe, G. H. Jeut, Eds. (Verlag Marie Leidorf GmbH, 2010), pp. 239–250.

[126] M. Roslund, “Crumbs from the rich man’s table: Byzantine finds in Lund and Sigtuna, c. 980–1250,” in *Visions of the Past: Trends and Traditions in Swedish Medieval Archaeology*, H. Andersson, L. Ersgård, P. Carelli, Eds. (Riksantikvarieämbete, 1997), pp. 239–297.

[127] T. Zachrisson, “Sigtuna: An urban hub in the Viking world, and its roots,” in *Viking Encounters: Proceedings of the eighteenth Viking Congress, Denmark, August 6–12, 2017*, A. Pedersen, S. Sindbæk, Eds. (Oxbow Books Ltd., 2020), pp. 268–285.

[128] T. Zachrisson, “Vikingatida Sigtuna, både nära och fjärran,” in *Tidens landskap : En vänbok till Anders Andrén*, C. Ljung, A. Andreasson Sjögren, I. Berg, E. Engström, A.-M. Hållans Stenholm, K. Jonsson, A. Klevnäs, L. Qviström, T. Zachrisson, Eds. (Nordic Academic Press, 2019), pp. 228–230.

[129] S. Tesch, “Skiftet och Sigtuna: Hybriditet och motstånd som en del av Mälarområdets kristnande,” in *Skiftet: Vikingatida Sed Och Kristen Tro: Ett Mångvetenskapligt Perspektiv På Kristnadsprocessen i Mälarområdet* (Artos, 2017), pp. 11–52.

[130] S. Tesche, “Sigtuna: Royal site and christian town and the regional perspective, c. 980–1100,” in *New Aspects on Viking-Age Urbanism c. AD 750–1100: Proceedings of the International Symposium at the Swedish History Museum, April 17-20th 2013.*, L. Holmquist, S. Kalmring, C. Hedenstierna-Jonson, Eds. (Archaeological Research Laboratory, Stockholm Univ., 2016), pp. 115–138.

[131] M. Roslund, “A geography of slavery: Ceramic networks and communities in the Lake Mälaren valley, Sweden c. ad 950 to 1150,” in *Vikings Across Boundaries* (Routledge, 2020), pp. 258–284.

[132] M. Roslund, “Tacit knowing of thralls: Style negotiation and hybridization among the unfree in 11th and 12th C. Sweden,” in *Archaeologies of Cultural Contact*, T. Clack, M. Brittain, Eds. (Oxford Univ. Press, 2022).

[133] A. Kjellström, “Bioarchaeological Aspects of the Early Stage of Urbanization in Sigtuna, Sweden,” in *The Bioarchaeology of Urbanization: The Biological, Demographic, and Social Consequences of Living in Cities*, T. K. Betsinger, S. N. DeWitte, Eds. (Springer International Publishing, 2020), pp. 119–145.

[134] C. Ljung, T. Zachrisson, A. Kjellström, På höjderna och i stadens utkant: Gravfält och gravgårdar i det äldsta Sigtuna. Fornvännen 2024, 199–223 (2024).

[135] M. M. Buś, M. Lembring, A. Kjellström, C. Strobl, B. Zimmermann, W. Parson, M. Allen, Mitochondrial DNA analysis of a Viking age mass grave in Sweden. Forensic Sci. Int. Genet. 42, 268–274 (2019). 31442669 10.1016/j.fsigen.2019.06.002

[136] V. Alexandersen, E. Iregren, L. Redin, “Nya kunskaper om livet på storgården” in *Västerhus: Kapell, kyrkogård och befolkning*, E. Iregren, V. Alexandersen, L. Redin, Eds. (Kungliga Vitterhets- historie- och antikvitetsakademien, 2009), pp. 244–248.

[137] E. Iregren, V. Alexandersen, L. Redin, *Västerhus: Kapell, Kyrkogård Och Befolkning* (Kungl. Vitterhets historie och antikvitets akademien, 2009).

[138] V. Alexandersen, E. Iregren, Westerhus–Børnenes tænder. Hikuin 27, 203 (2000).

[139] T. Swärdstedt, *Odontological Aspects of a Medieval Population in the Province of Jämtland, Mid-Sweden* (University of Stockholm, 1966).

[140] B. Helgesson, C. Arcini, A major burial ground fiscovered at Fjälkinge: Reflections of life in a Scanian Viking Village. Lund Archaeol. Rev. 2, 51–61 (1996).

[141] B. Helgesson, *Järnålderns Skåne: Samhälle, Centra Och Regioner* (Lund Univ., 2002), vol. 38.

[142] O. Svensson, “Nämnda ting men glömda: Ortnamn, landskap och rättsutövning,” thesis, Linnéuniversitetet, Växjö (2015).

[143] C. Ahlström Arcini, T. D. Price, S. Hyll, *The Viking Age: A Time of Many Faces* (Oxbow Books, 2018).

