# Supplementary material for: Equal in death: Ancient genomic analysis of children’s early Christian burials
Source: Sci Adv. 2026 Jul 10;12(28):eaeb8588. doi: 10.1126/sciadv.aeb8588 (PMC13353417; doi:10.1126/sciadv.aeb8588)
Supplement: Supplementary file 1 — Supplementary Text Figs. S1 to S10 Table S1 Legends for tables S2 to S15 References [file sciadv.aeb8588_sm.pdf]

Science Advances
AAAS

# Supplementary Materials for

## Equal in death: Ancient genomic analysis of children's early Christian burials

Maja Krzewińska *et al.*

Corresponding author: Maja Krzewińska, maja.krzewinska@arklab.su.se

*Sci. Adv.* **12**, eaeb8588 (2026)
DOI: 10.1126/sciadv.aeb8588

**The PDF file includes:**

Supplementary Text
Figs. S1 to S10
Table S1
Legends for tables S2 to S15
References

**Other Supplementary Material for this manuscript includes the following:**

Tables S2 to S15

**Supplementary Text**

1. Theoretical background

1.1. Sex assessments of subadults

The term "sex" pertains to the biological characteristics of an individual, though its definition is not always straightforward. It may refer to chromosomal composition, internal and external genitalia, variations in hormone levels, or reproductive capacity. The gametes decide the chromosomal sex of a child and in the fifth to sixth week in utero the genitals start to develop. Though slight differences in ossification rates or timing of mineralisation in teeth exist during the foetal period with girls developing earlier than boys (*89*, *90*), the difference in osteological appearance between the sexes is small. After birth, girls continue to mature at an earlier stage even though boys may have slightly larger bones (*90*). Similarly, the growth spurt at puberty generally starts earlier in girls. Hence, growth involves changes in both size and maturity, and by that morphology, all of which usually correlate with age. However, genetic and environmental factors may lead to large individual differences. In association with puberty and sexual maturity the hormonal changes result in the development of secondary sex characteristics (i.e., physical features not involved in reproduction). The most prominent sexually dimorphic traits of the skeleton are related to pelvic features (e.g. (*2*, *91–93*)) and the morphology of the skull (e.g., (*2*, *94*, *95*)). Since the levels of sex hormones are low before the onset of puberty, the skeletal dimorphism between boys and girls is minimal. Although, a range of studies exists within bioarchaeology where attempts have been made to assess sex in juveniles (e.g., (*4–7*)), the success rates have been poor, or mediocre at best (*96–100*). An additional problem in archaeology is the fragile nature of juvenile skeletons which are easily affected by taphonomic agents and hence often found in a fragmentary state. The numerous difficulties related to the classification of physical characteristics of juvenile skeletons have sometimes led to the usage of external factors associated with a specific gender (e.g., grave goods), to separate boys and girls (e.g., (*9*, *101*)). Gender needs not to correspond with biological sex, and gender is seen as a social construction and may vary in time and between societies (however, some researchers believe that sex is also a construction, see Sørensen 2000 (*102*)). Although the procedure is understandable at times, sexing via material culture risks obscuring variations in socialization processes for individuals of different biological sexes, or reinforcing preconceptions about past populations. The possibility to identify the chromosomal sex of young individuals will not end discussions about gender versus sex in the past, but it will bring new data to the debate (see e.g., (*103*)).

*Childhood Archaeology and Socialization Processes*

Children are often underrepresented in archaeological studies due to the poorer preservation of juvenile remains, as their bones are more susceptible to diagenesis compared to adults (*104*). Additionally, there are challenges in reliably determining sex and identifying trauma in the more pliant bones of subadults, which has led to these remains being considered less informative for reconstructing the past (*3*, *105*). However, childhood is increasingly recognized as a social construct that reflects the values and perceptions of contemporary society (*106*), making it a crucial aspect of understanding social organization. Social age, or the perception of a child's role and their transition to adulthood, can provide important clues when inferred from grave contexts (*107*). Although it is challenging to define specific stages of social age for Viking Age and early medieval societies due to a lack of historical records and potential regional variation, some scholars suggest that individuals might be considered adults from the

age of 12 (*108–110*), while provincial laws indicate regional differences, with adulthood sometimes being recognized as late as age 16 (*110*). In medieval societies, the transition to adulthood appears to have been more individualized (*29*), though a child was considered religiously mature by the age of seven (*20*). Historical records, such as miracle stories, suggest that children may have entered the workforce as early as age nine (*111*), and girls might have married between the ages of 14 and 18 (*112*), indicating multiple thresholds in the experience of childhood during this period.

1.2. Medieval Scandinavian burial traditions and regulations

With few exceptions, there are no written formal regulations for the execution of funerals or mortuary practices from the Viking Age in Scandinavia (*13*). The period is characterized by a large contemporary variation in burial expression, from funeral pyres in burial mounds to simple inhumation burials, with or without visible markings above ground. At larger Viking hubs there was of course a greater inclusion of more exclusive elements such as chamber tombs, and some regionality in practice related to time may be discerned, but diversity is also found at small local burial sites (e.g., (*14*)). At least personal items followed the deceased in most graves, and a few were magnificently furnished. The size of the cemeteries varies from a few graves to thousands, related to the constellation of the people who inhabited the area and the function of the site. Concentrations of graves in groups, especially in association with single farms, have, among other things, been interpreted as family burial grounds (*113*). Most graves, whether funeral pyres or inhumations, are arranged for one individual but for both categories there are many examples of double graves. Among these, there are cases where one of the individuals have been ritually killed and interpreted as a person of inferior status (*16*). As Christianity spread through Scandinavia in the end of the 10th and in the 11th century, the mortuary practices changed, and became more uniform. In the intermediate stage, before the erection of churches, people were buried unburned at their homesteads and there was also a re-use of older burial mounds (*17*, *18*, *114*). Historical records are missing but there is no reason to believe that the arrangements of graves or the funeral act that was initially conducted by foreign missionaries, should have differed from the liturgical practice in Christian Europe (*19*). Ideally, the ritual started with the dying receiving communion, later the body was washed and clothed at home. Specific prayers and songs were performed during mass in church and at the grave. The burial rite could take place on the same day the individual died but only baptized were interred in the Christian cemetery (i.e., not even hastily deceased infants were allowed to get buried on consecrated ground) (*19*, *20*). The characteristics of a Christian grave of the time were: single east-west oriented burials, with a supine body lacking grave goods except for items related to the dress or shroud of the deceased, and with no or simple markings above ground (e.g., (*18*, *21*)). The preparation of the medieval grave and body was related to the importance of the resurrection (*22*). However, it is clear that some theologians already in the 9th century considered the burial procedures were primarily acts of comfort for relatives (*19*). In the absence of contemporary records, the attitude towards individuals sharing the same grave is difficult to ascertain, but there are 4th century texts in which it is assumed that married couples are buried together (cited literature in (*19*)).

The location of graves was linked to liturgic and ideological principles, reflecting ecclesiastical injunctions and the secular social order of the time (*19*, *23*). A social zoning system existed where mainly clergy but also the lay elite were buried near the church altar, and people

with lower social status in descending order further from the spiritual centre in the churchyard. The Norwegian Borgarthings law, for instance states that slaves were to be buried in the outskirts of the churchyard (*24*). Additionally, the Eidsivathings law (11th-12th century), advocated that the burials were to be segregated by sex with women buried to the north and men to the south (*20*). Churchyards that validate these laws have been demonstrated in all Scandinavian countries (e.g., (*20*, *23–26*)), although there seem to exist regional differences for the implementation of sex segregation. Concentrations of child burials at many churchyards, near the church (often around the choir), also implies that social factors related to age could govern the location of a grave.

### 1.3. Multiple burials

Deviations from the established practices have the power to highlight subtle details reflecting individual- and social attributes and concerning the inhumed individual's surroundings. Multiple burials are examples of such unusual funerary practices and in comparison, to single graves, the multiple burials complement our understanding of the complex social interactions since they deviate from the norm. Such burials are present throughout medieval Scandinavia (*30*). In its simplest form, a multiple burial (also plural burials or collective burials) is a funerary structure where two or more individuals were inhumed (*56*). However, there are many types and subcategories of this kind of burials, including mass-graves or family tombs, where deceased representing more than one generation may have been interred. Miniaci (2019) has discussed the inconsistent terminology for these graves, and highlighted time and architectural space as decisive factors. In the current study most of the graves fit the definition that Miniaci's name primary simultaneous multiple burial. This denotes a grave where the individuals are buried at the same time without having been moved secondary. Additionally, there are agglutionating burials, which are graves that involve a female with a fetus/newborn
(*56*). This latter type of burial need not be considered true multiple burial as it is not clear that the contemporary society knew that a female was pregnant, and that this constellation could be seen as a unit on its own (*56*). Burials containing women with small children do not seem to be different to other multiple burials e.g., in terms of sex distribution. The majority follow the rule of keeping same sexes together, but there are exceptions.

All graves in the present study have, by archaeologists, been considered as simultaneous burials (i.e., including individuals that died at, or around, the same time of an unknown reason). However, it cannot be completely ruled out that there are cases of sequential multiple burials (*56*), where the graves of a single individual have been reopened without a major time gap for another individual to share the same grave. The individuals included in the single-burial category were not selected based on any archaeological criteria but were simply those for whom suitable skeletal material, specifically mandibles containing intact teeth, was available for sampling. Their spatial proximity within each cemetery was therefore not used as a criterion, and the individuals represent a random subset of single interments as preserved at each site.

### 1.4. Biological kinship and social relations of the era

Commonly, when no clear context for multiple inhumation is available, kinship has often been assumed to be the grounds for such funerary practice. Kinship is a complex notion with a broad definition which is heterogenous between ranges of disciplines. It is often defined as biological affinity, resulting in exclusion of other than biological forms of relationships such as

e.g., adoption or alternative types of social organisation including marriage or emancipation. Thus, biological relatedness and social affinity are different aspects describing mutual relations between humans. The distinction between the two types of relations can be problematic in the archaeological record (*33*). Without genetic data a direct biological kinship can only be inferred reliably from a burial context in exceptional cases, for example, upon discovery of female-foetus burials. But even seemingly obstetric burials do not necessarily represent biological kinship. It has been argued that in a number of such burials, infants may have been inhumed at a later stage, especially when the child is no longer a neonate (*57*). Burials in which children were interred with adult males further complicate the matter. Children may have been placed in adult burials as a result of coinciding death, rather than kinship relation - a tradition known from the Anglican Church practices (*58*). Essentially, only cases in which a female was buried with an infant unequivocally located in utero can testify to direct kinship.

Whenever a deviant mortuary practice of this type cannot be linked to biological kinship or is only inferred based on assumptions, it could reflect other social relations or the social perception of the deceased. Adult couple burials with a male and a female have been thought to represent a marital union, or master/mistress and servant (*115*, *116*). However, it has been shown that same sex combinations (e.g., females or males only) are fairly common in medieval Scandinavian multiple burials (*30*, *34*). If a biological kinship can be ruled out, the affiliation between these individuals must have been of a different kind and may represent special types of bonds between the deceased such as: common background (ethnicity), lifestyle or work environment.

Though, people could live in nuclear family constellations (i.e., parents with biological or adopted children), most households of the era were probably larger than that, involving older relatives, employees and slaves (*59*). Even though kinship was important, previous notions regarding the strong clan society (Sw. ättesamhälle) in Viking and Early Middle Age Scandinavia have been modified by scholars during later years (*60*, *61*, *117*). Instead of a strictly blood-related group of people, researchers have shown that what constituted a clan was much more fluent and varied, and that there was an element of choice for who would be included. Nevertheless, with the rise of Christianisation, the church seems to have gradually influenced the provincial laws, and with that came an "ecclesiastical definition of kinship" (*60*). In either case, marriage was an important tool for economic or political alliances, but there existed also other forms of non-biological relationships and cohabitation. Another social bond that has been highlighted by historians during later years is friendship alliances among adults (*59*). The importance of having a reliable loyal friend facilitated everyday life, not least in conflict situations, and different types of friendship are enumerated in Norse sources (*59*). Except for marriage, concubinage could create and strengthen ties between groups (*61*), and many children born out of wedlock were cared for. According to provincial laws in Scandinavia and in Iceland these children could inherit from their parents (*61*, *63*, *64*), and there exist several categories of extramarital children in Danish and Norwegian laws (*64*). The relationship between an adult and a child could be equally complex to that of adults, and be based on other ties than blood since fostering and apprenticeships was common (*62*, *118*). The custom where children were sent by the biological parents to be reared by other families, not necessarily kindred, seems to have been fairly common (*65*). It is also likely that younger children could develop bonds with wet-nurses and hired caretakers (*119*).

Regardless of what social ties people had with each other, the presence of a multiple grave indicates that the buried must have died close in time. Also, it can never be ruled out that some burial compositions may have been purely coincidental and of a pragmatic nature. Warfare, epidemics and accidents could result in instances of simultaneous death, but in the absence of clear signs of violence or demographic patterns consistent with epidemics (e.g., (*70*)), and when dealing with fairly homogenous burials, the mechanism and manner of death is less clear.

## 2. Materials

### 2.1. Sigtuna

Sigtuna, the oldest medieval town in Sweden, was founded in the Mälaren Valley at the end of the tenth century. It is now debated if the centre was built on virgin soil (*120*), nevertheless it rapidly showed an urban character with a regular structure of dense plots and wooden trackways. Researchers agree that the town was established on royal initiative and thus, with its strategic location, became an important political and administrative centre, involving Sweden's first coinage among other things (*121–124*). Itinerant or resident craftsmen and merchants worked in the many workshops. Historical documentation, iconography and artefacts from the black soil, reveal close contacts with the east, especially Kiev and Novgorod, but also Denmark and the North Sea Empire, as well as to the German and Frisian area (*124–128*). At the time of the foundation of Sigtuna, Christianity was on the rise and with time several stone churches were erected. With the help of Christian missionaries from both England and the Hamburg-Bremen archdiocese the town also became a religious centre, and in the 1100s an episcopal see was established (*129*, *130*). The composition of the urban population has been discussed. Some researchers state that Sigtuna's plots were managed by employees of an elite that lived on farms in the surrounding landscape (*124*, *130*). Other researchers put forward, based on the deviating material culture in the town towards the countryside, that Sigtuna mainly housed long-distance guests who could have been more or less permanent visitors (*131*, *132*). Bioarchaeological analyses suggest an intermediate explanation (*133*), since genetics and strontium values from buried people have shown that the composition of the population was heterogeneous, with people from both the immediate area but also, from other parts of Europe (*41*).

The early Christian burial customs varied in both location and ritual. The urban dead were interred in diverse settings, including stone grave constructions on elevated terrain, flat-ground cemeteries near settlements, and early churchyards. Except for a few documented pre-Christian burials, the rest are unmarked east-west oriented graves, lacking burial offerings and with a Christian character. The oldest graveyards (c. 10th to 11th century), consisting of groups of 54–60 graves, are located in a semicircle around the town and lack an attached church (*134*). The later stone churches (c. 11th to 12th century) encompassed churchyards seemingly governed by the strict medieval burial regulations (*133*). Up until 2016, more than 1000 graves have been excavated of which approximately 850 have a well-documented archaeological context (*133*). By 2010, 17 multi-person graves, with 35 buried individuals, from four churchyards (Church 2 in the St Nicholas block, St Laurence's, St Olaf's, and Church 3 in the Magistern block) had been discovered in Sigtuna (*34*). In the churchyard of St Laurence's there is also a mass grave with 20 individuals, not included in this study (*135*).

## 2.2. Västerhus

In the 11th century, a magnate's farm was established at Frösön in the province of Jämtland, in northwest Sweden (then Norway). It has been suggested that the estate had a fiscal role and that the lord of Västerhus functioned as the representative of the king (*136*). In association with the manor was a chapel and a churchyard which were used by the wealthy magnate family and people working at the estate during the eleventh to the late fourteenth-century (*27*, *137*). The site was excavated during the 1940-1950s, and the investigated churchyard revealed 371 skeletons whereof 226 are individuals below 18 years of age (*137*). Almost all the buried adults were segregated by sex so that women (n = 80) had been buried to the north and men (n = 76) to the south of the church (*25*), although some young individuals have undergone reassessments (*137*). The children have been thought to follow this spatial distribution (*25*, *138*). Hence, the sex distribution and signs of a distinct social hierarchy in location of burials (*25*, *27*, *139*) follow the Scandinavian burial regulations. The recording of the graves during the excavation did not always follow the professional standard for archaeologists and the context is due to this sometimes difficult to evaluate. However, a conservative estimate has been that at least 34 multiple burials involving 91 individuals, were located at the cemetery (*30*).

## 2.3. Fjälkinge

In the north-eastern province of Scania (then Denmark), in the parish Fjälkinge, a Viking Age cemetery (AD 900-1050) was excavated in 1990 (*140*). The site has been interpreted as being one of at least two cemeteries belonging to a Viking Age village in the rich agrarian landscape which seems to have been cultivated for a long time (*140*, *141*). Fjälkinge has roots in the late Iron Age and was important during the Viking Age. According to Ola Svensson (2015), the thing-site at Fjälkinge exemplifies the strong spatial link between early legal assemblies and monolithic rock formations, with prehistoric sacrificial finds indicating the site's long-standing special status (*142*). In historical times, Fjälkinge was one of Scania's largest villages, housing around 50 farms. In total 121 graves containing 128 individuals were documented, whereof 24 were men, 24 women and 80 children. Some of the graves contain burial goods and are oriented north-south, others lack artefacts and have an east-west orientation, implying a Christian influence. The position of the bodies varies with a majority of supine burials, but there are also bodies placed on the side, at least one prone individual, and bodies in hunched positions. The age distribution showed that 81.2% of all children were below the age of 1 year (*140*). Among the adults, the sex distribution was even and an unusual number seem to have been over 60 years old (*143*). At the site there are four possible double graves (graves 314, 399, 776, 952) (*140*). There are also seven superimposed burials with one grave buried on top of the other suggesting a possible relationship between the individuals (graves 215, 360, 952, 952, 304, 428, 65) (*143*).

## 2.4. Generated data

Originally, we set out to sample 130 individuals from multiple burials in three locations: Västerhus, Fjälkinge and Sigtuna. We have used genetic data from 118 individuals from multiple burials, of which three are shared with other studies (win001, win002, wes007), and present additional genomic data (n = 25) from non-multiple burials from the same localities. Not all individuals were successfully retrieved from the collections. In the Västerhus collection, some of the individuals were missing: all three infants from burial 145 (no individuals from 145 left to analyse thus excluding burial 145 altogether), the infant from burial 147 (only the adult available

thus a single burial), in burial 157 one infant was missing (thus 5 out of 6 individuals in that burial were available), in burial 167 the foetal bones were missing (thus 3 out of 4 individuals in that burial were available). Furthermore, some samples failed before sequencing (n = 6) leading to the exclusion of complete (Sigtuna Id97096: one out of two individuals in one burial, thus making it a single burial) or partial (Västerhus burials no 25, 50, 72, 102 - two out of four individuals) burials from the analyses. Thus, for the main part dealing with sex and age distribution, all remaining individuals were used (118) of which 116 were in 50 multiple burials. For kinship (including uniparental markers) and population genetic analyses, only those with a genome coverage above 0.01x were included, leading to the exclusion of 21 individuals (11 from Västerhus, 2 from Fjälkinge and 8 from Sigtuna). We have also generated reference data from 25 additional individuals, and collected further 23 published genomes to be used as reference (table S1: Ind No 119-165 and 74).

2.5. IBD network analyses

To assess levels of background relatedness across sites, identity-by-descent (IBD) analyses were conducted to capture more distant genetic relationships beyond the resolution of standard kinship estimation methods. Network analyses were used to visualise IBD sharing among individuals. The networks were generated in Gephi 0.10.1 (*85*) using the Fruchterman–Reingold layout algorithm (*86*). Only IBD segments with a minimum length of 8 cM were included. Networks were visualised in two ways: (A) based on weighted connections representing the number of shared IBD segments ≥8 cM (fig. S4) and (B) based on the maximum shared IBD segment length between individual pairs (fig. S5). In the network visualisations, nodes represent individuals (green: single burials; pink: multiple burials), node size is proportional to the number of connections, and edges represent shared IBD segments. These analyses indicate substantially higher levels of background relatedness at Västerhus compared to Sigtuna, consistent with differences in shared ancestry between the sites. This pattern likely reflects differences in site character, such as rural versus urban contexts, rather than population-level divergence.

2.5. Population genomic analyses and downsampling

To explore the genetic affinities of the newly sequenced individuals, we conducted a series of $f_3$ and $f_4$ statistics analyses using a broad reference panel. Specifically, we calculated individual-level $f_3$ statistics for newly generated samples against a subset of reference populations from AADR v62 (*82*) (table S12), and individual-level $f_4$ statistics using both modern and ancient reference panels (table S13). Lowest coverage and related individuals were excluded from those analyses. We additionally visualized pairwise $f_3$ statistics using multidimensional scaling (MDS) in R using the cmdscale function in R (v. 4.4.3) (fig. S6 to S7). Across the $f_3$, $f_4$, and MDS analyses, we observed limited genetic structuring: most individuals form a single, relatively tight cluster. The few outliers correspond either to previously identified individuals (*35*, *41*, *69*) or to samples with relatively low coverage. The only newly detected outlier is mbv190, who is the father of previously identified outlier individual wes056 and therefore expected to show a close but distinguishable genetic position (*35*).

To ensure that group $f_3$ statistics were calculated from a dataset with balanced sample sizes and minimal influence from outliers or related individuals, we applied two complementary downsampling strategies. First, we removed all closely related individuals (retaining the higher-coverage sample from each pair), excluded all capture-enriched and low-coverage individuals,

and merged previously identified outliers into their appropriate population groups. This procedure yielded a dataset of 60 individuals from Västerhus, 54 from Sigtuna, and 8 from Fjälkinge, which we visualized using MDS plots (fig. S8). Although this approach provided an initial overview of group-level relationships, with Fjälkinge being closer to Viking and Early Viking individuals from southern Sweden, other observed group-level differences likely reflect residual effects of within-group outliers and uneven sample sizes.

Therefore, in a second step, we further downsampled the dataset by excluding all population groups with fewer than five individuals and retaining only the ten highest-coverage individuals from each remaining group. The resulting dataset was then visualized using MDS based on $f_3$ statistics in R (fig. S9). This seems to confirm that Fjälkinge is closer to Viking and Early Viking Danish groups while Västerhus resembled more central Sweden Vikings. Sigtuna individuals exhibit broad variation and therefore Sigtuna falls between Scandinavian and the Baltic and Russian Vikings.

Finally, to address the uneven sample sizes and varying chronological periods between the three studied sites, and to reduce the impact of "background relatedness" on population genomic analyses, we implemented a downsampling strategy based on Identity-by-Descent (IBD) sharing to compare the tree sites. At sites like Västerhus, elevated background relatedness - evidenced by extensive sharing of 8cM IBD segments (figs. S4 to S5) - could potentially influence genetic affinity. To ensure that our inter-site comparisons were not driven by a few highly interconnected families, we performed a more stringent subsampling based on IBD sharing patterns:

First, we selected only individuals sharing zero IBD segments (≥8 cM) with any other individual in the dataset. This resulted in a limited sample size: Sigtuna (n = 7), Fjälkinge (n = 3), Västerhus (n = 1), and Lund (n = 0). As this stringent subset likely over-represented "biological outliers" or recent migrants, we expanded the filtering and selected individuals sharing no more than two IBD segments of ≥8 cM with others. This increased the sample to Sigtuna (n = 40), Fjälkinge (n = 6), and Västerhus (n = 7). This subsample excludes close kin while retaining individuals who represent the broader shared ancestry of the local communities. We then filtered out the group $f_3$ statistic results calculated between the pairs of selected individuals and used the values to prepare a distance matrix derived as $1\text{-}f_3$. The matrix is visualized via classical Multi-Dimensional Scaling (MDS) using the cmdscale function in R (v. 4.4.3) (fig. S10). This analysis confirmed that Sigtuna individuals, from both single and multiple burials, exhibit a broad genetic clustering that also includes individuals from Västerhus. In contrast, the Fjälkinge individuals fall outside this range, indicating distinct genetic affinities.

**Provenance of Human Remains**

The skeletal material analyzed in this study was sampled between 2014 and 2018 by JS, MK, and AK. Samples were sourced from the osteological collections housed at the Lund University Historical Museum, Sigtuna Museum, and the Swedish History Museum (*Statens historiska museer*) in Stockholm. The authenticity of the remains was validated through a comprehensive review of original excavation records and archaeological reports, ensuring the direct association between the skeletal elements and their recorded provenance. The specimens were aged and dated based on their archaeological context, including stratigraphic evidence and burial typology established during the primary excavations. In addition, a subset of individuals has been

radiocarbon dated ($^{14}C$) to further refine the chronological framework. Following the sampling procedures, all skeletal material was returned to the respective curatorial institutions. The remains are permanently housed within these museums and remain accessible to the research community, subject to the standard access and ethical protocols of the curating institutions.

## Supplementary Figures

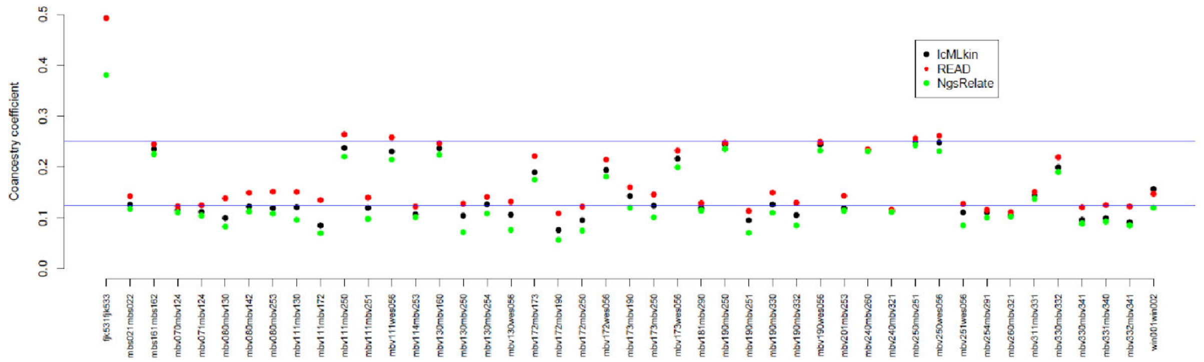


**Fig. S1**.

**Methodological concordance of kinship estimates.** Kinship estimate overlap between the used methods, i.e., READ (*44*), lcMLkin (*45*), and NgsRelate v2 (*46*, *47*).

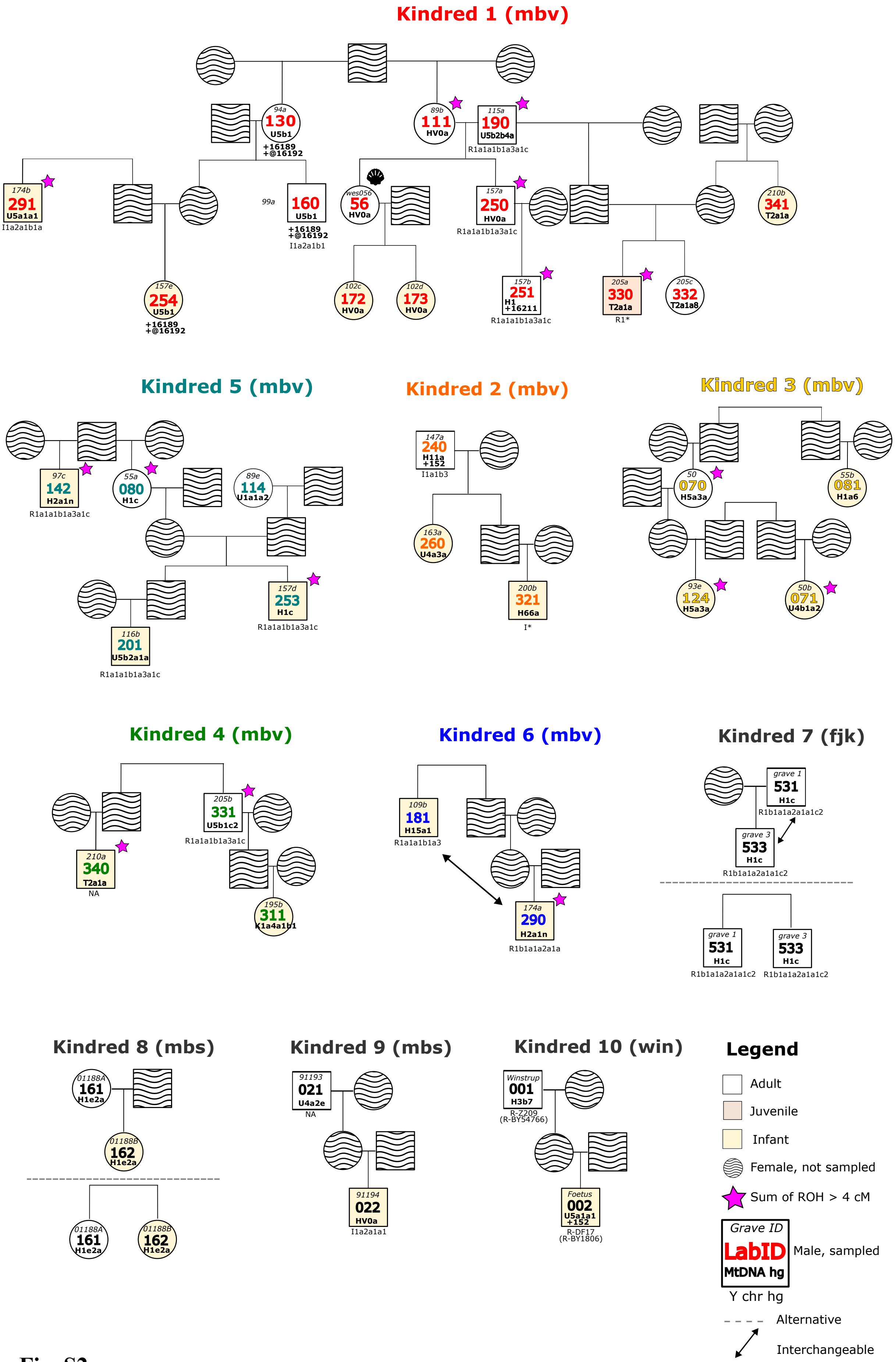


**Fig. S2.**
**Pedigree reconstructions and ROH distributions.** Detailed kindred information and pedigree reconstruction based on up to 2nd degree kinship as summarised in Table 2A and S9. Marked with a star are individuals with identified elevated levels of ROH fragments.

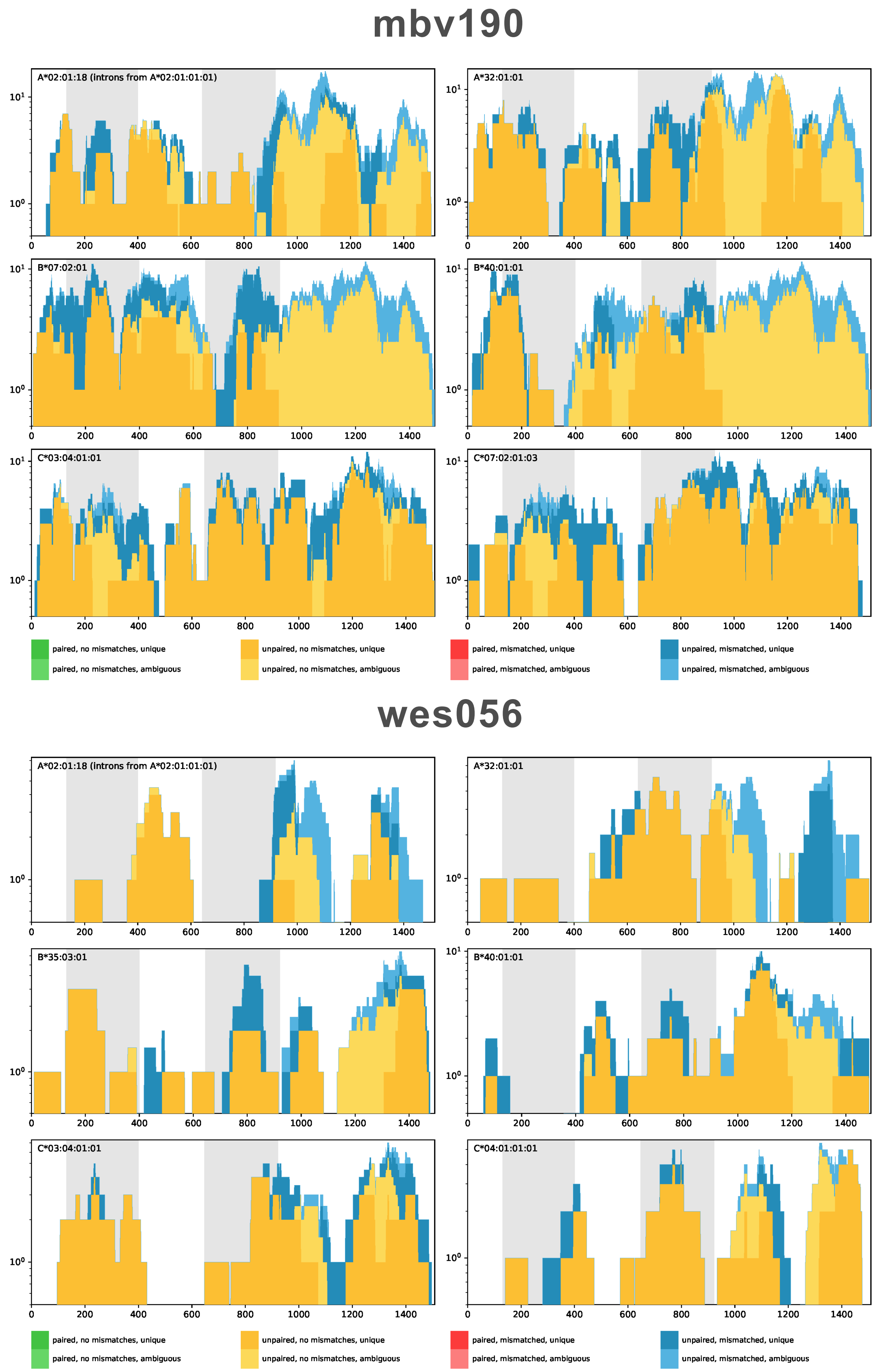


**Fig. S3.**
**HLA allele typing results.** Results of nf-core/HLAtyping Optitype allele calling for mbv190, mbv250, mbv251 and wes056.

## mbv250

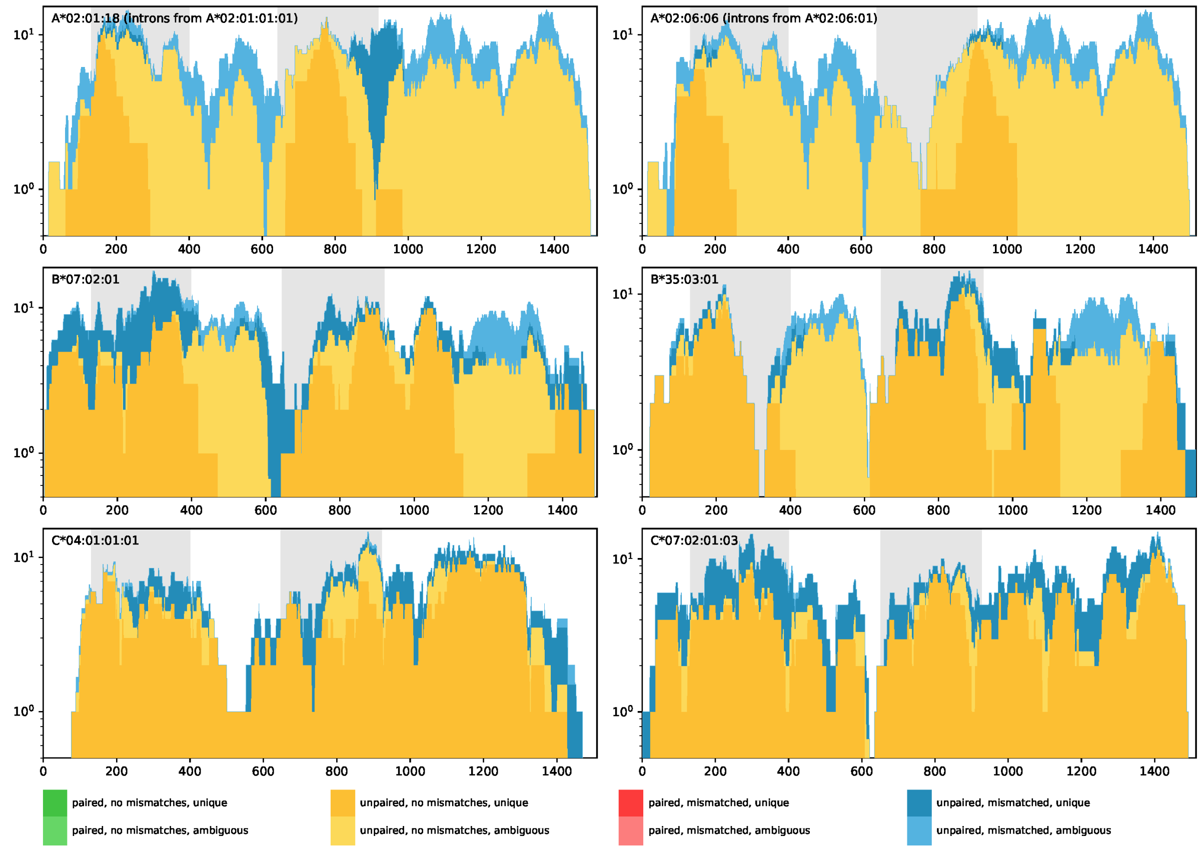


## mbv251

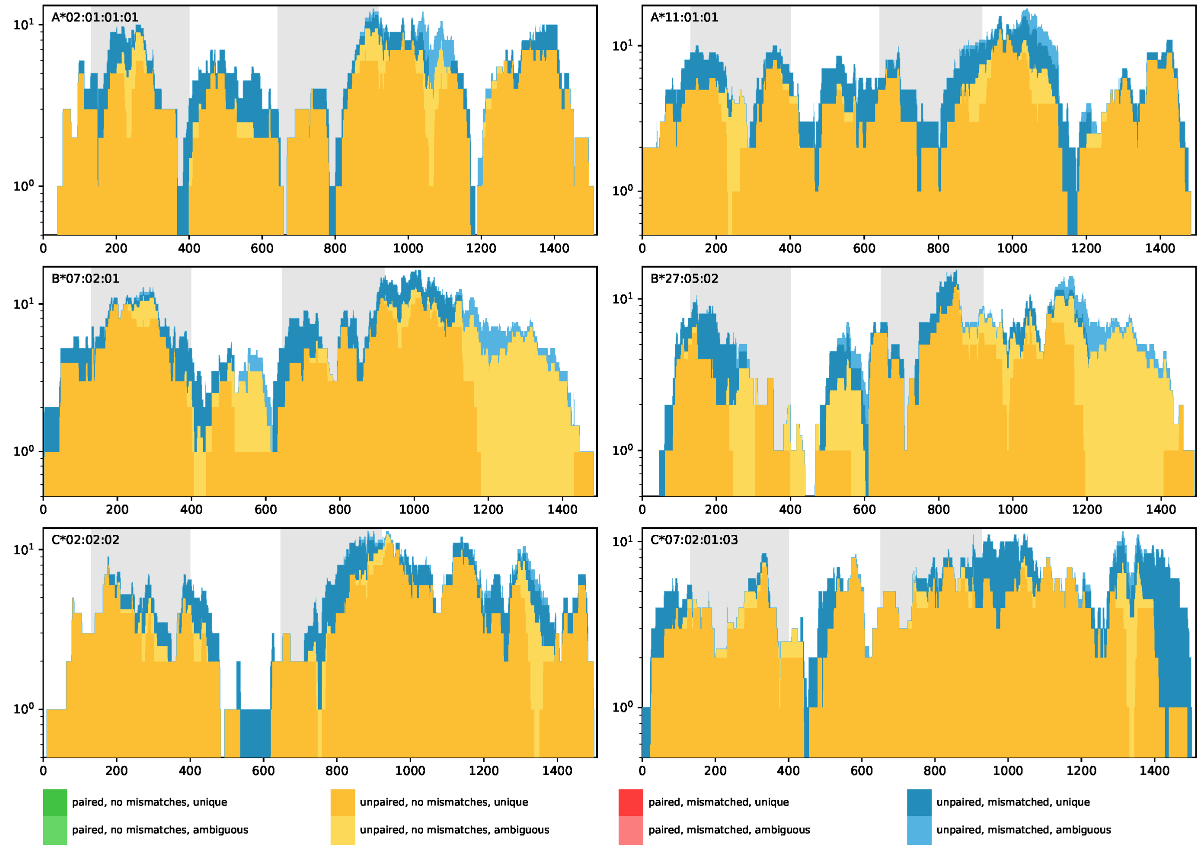


**Fig. S3 (continued).**
**HLA allele typing results.** Results of nf-core/HLAtyping Optitype allele calling for mbv190, mbv250, mbv251 and wes056 (continued).

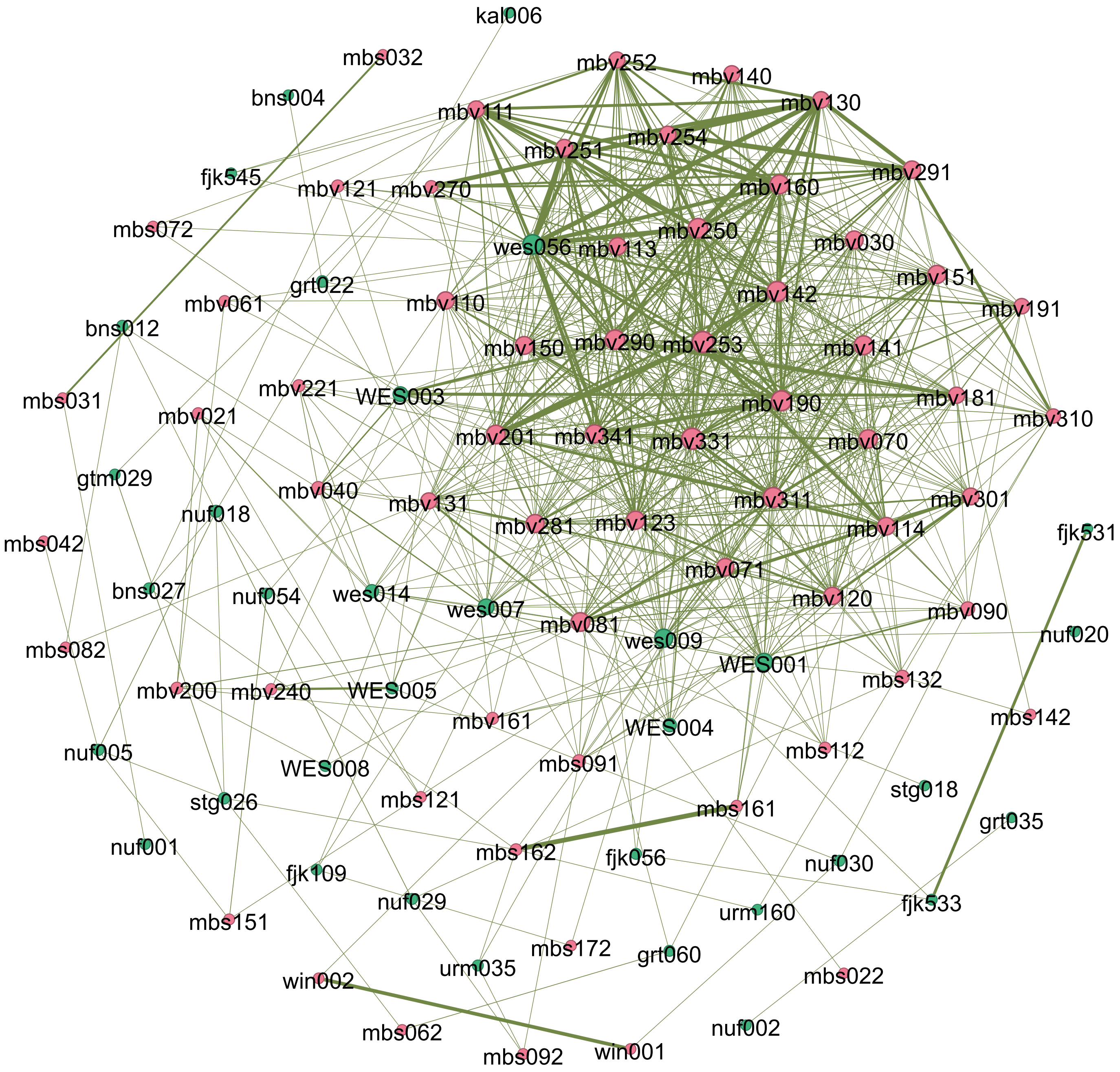


**Fig. S4.**
**IBD network by shared segment count.** Network of shared IBD segments showing all pairs of individuals in this study who share minimum one segment of ≥8 cM. The network was generated in Gephi 0.10.1 (85) using the Fruchterman–Reingold layout algorithm (86). Green nodes represent single burials, and pink nodes represent multiple burials. Node size is proportional to the number of edges connected to each node, and edge thickness reflects the number of shared segments (weight).

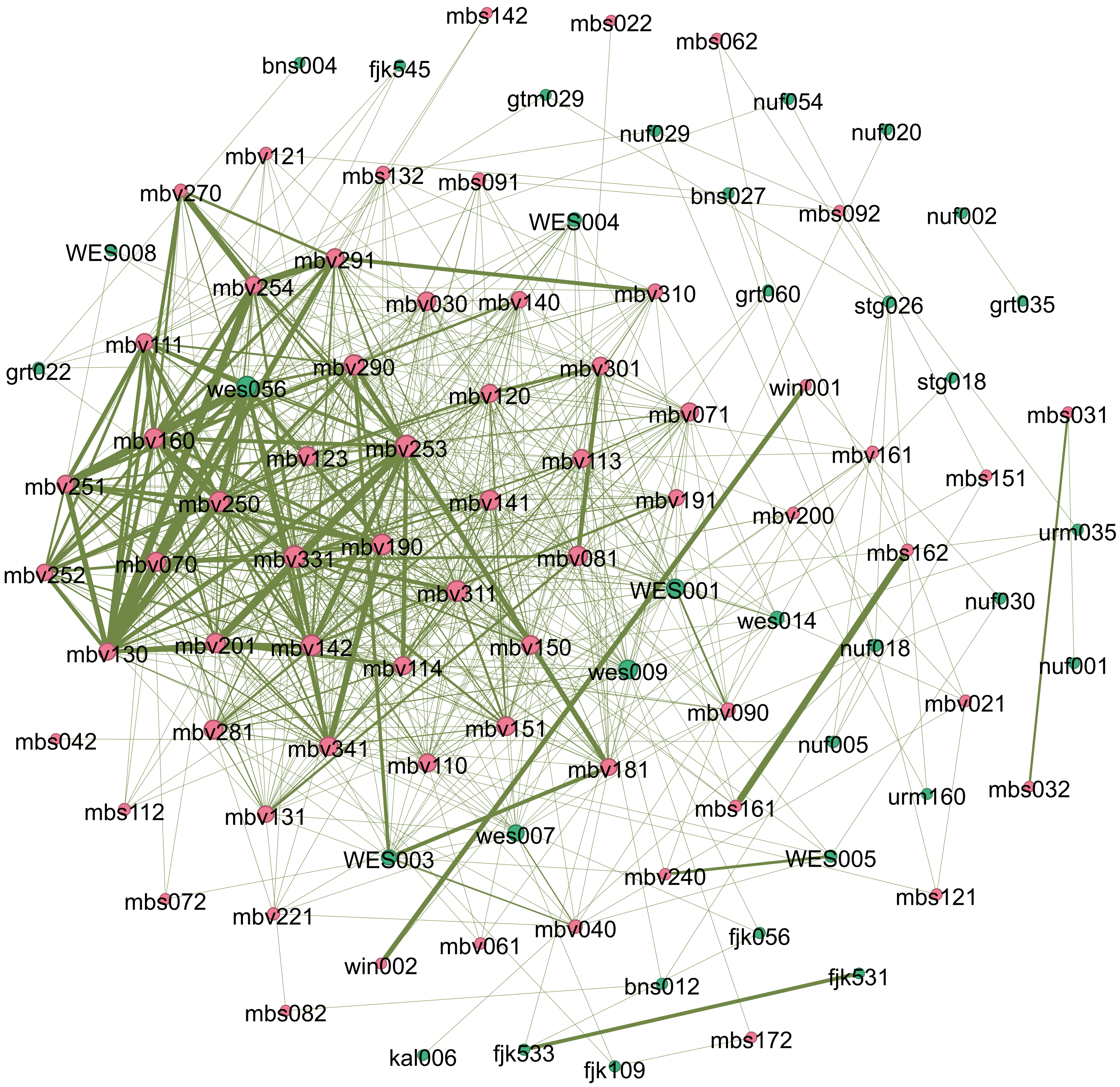


**Fig. S5.**

**IBD network by maximum segment length.** Network of shared IBD segments showing all pairs of individuals in this study who share minimum one segment of ≥8 cM. The network was generated in Gephi 0.10.1 (85) using the Fruchterman–Reingold layout algorithm (86). Green nodes represent single burials, and pink nodes represent multiple burials. Node size is proportional to the number of edges connected to each node, and edge width reflects the maximum IBD segment length shared between each pair of individuals (max).

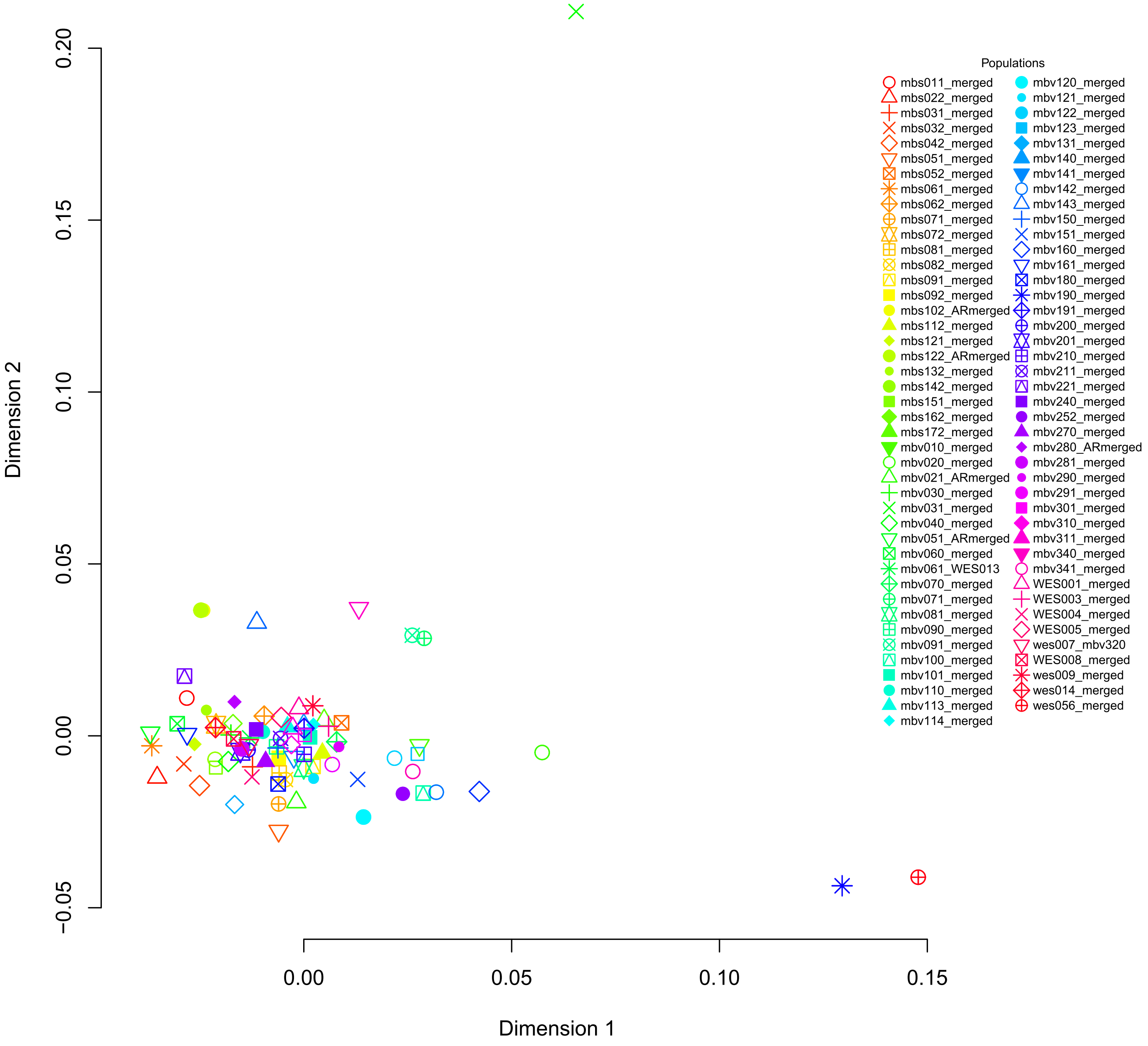


**Fig. S6.**
**Population continuity within the Västerhus site.** Multidimensional scaling (MDS) plot of pairwise f3 statistics for the newly sequenced individuals and previously published individuals from Västerhus (35). Most individuals form a tight cluster with minimal internal structure.

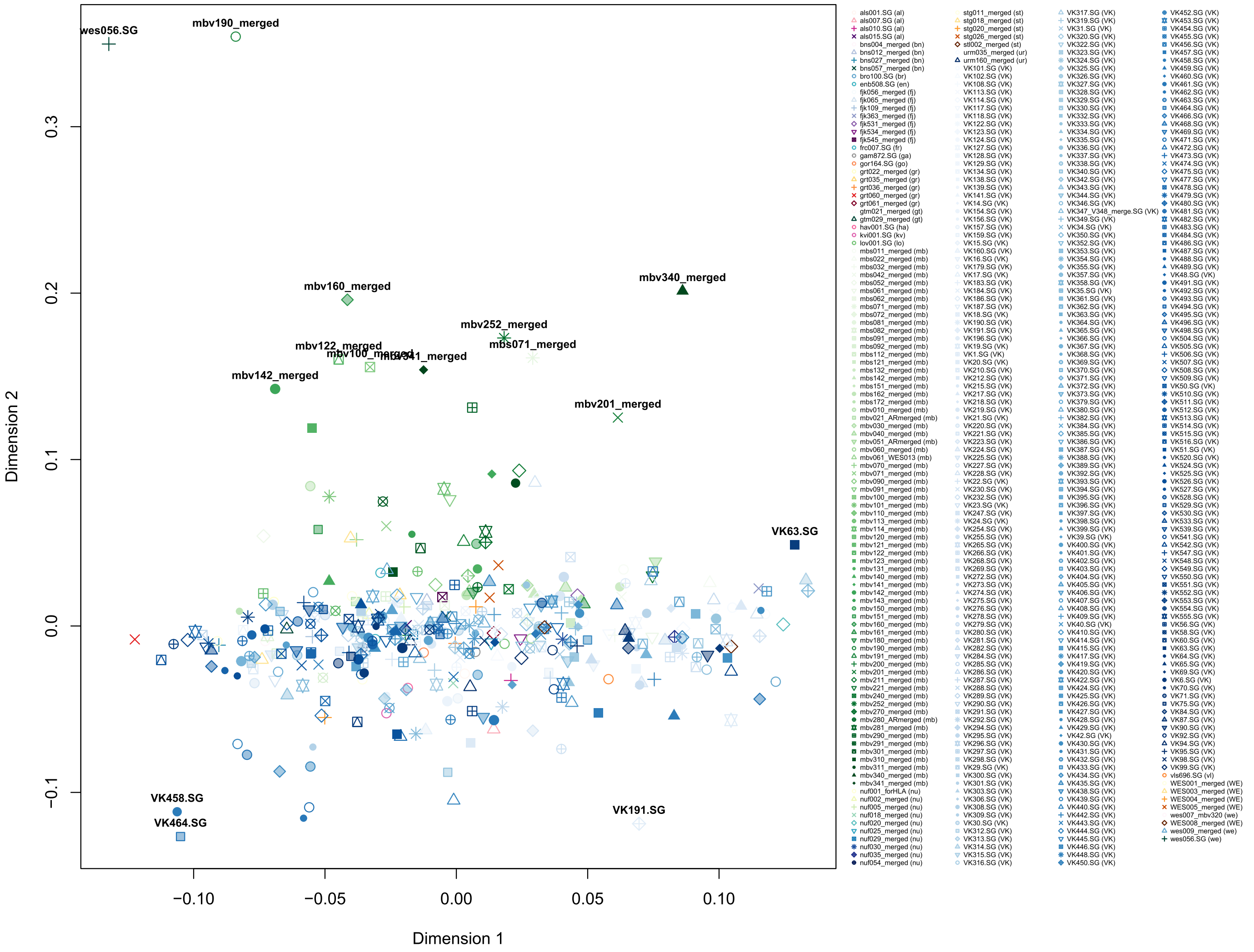


**Fig. S7.**

**Reference-based MDS of genetic affinities.** Second MDS representation of pairwise f3 statistics using a subset of reference populations. Limited structuring is observed, with most individuals clustering together and only a few known or low-coverage outliers separating from the main group (relates to table S12).

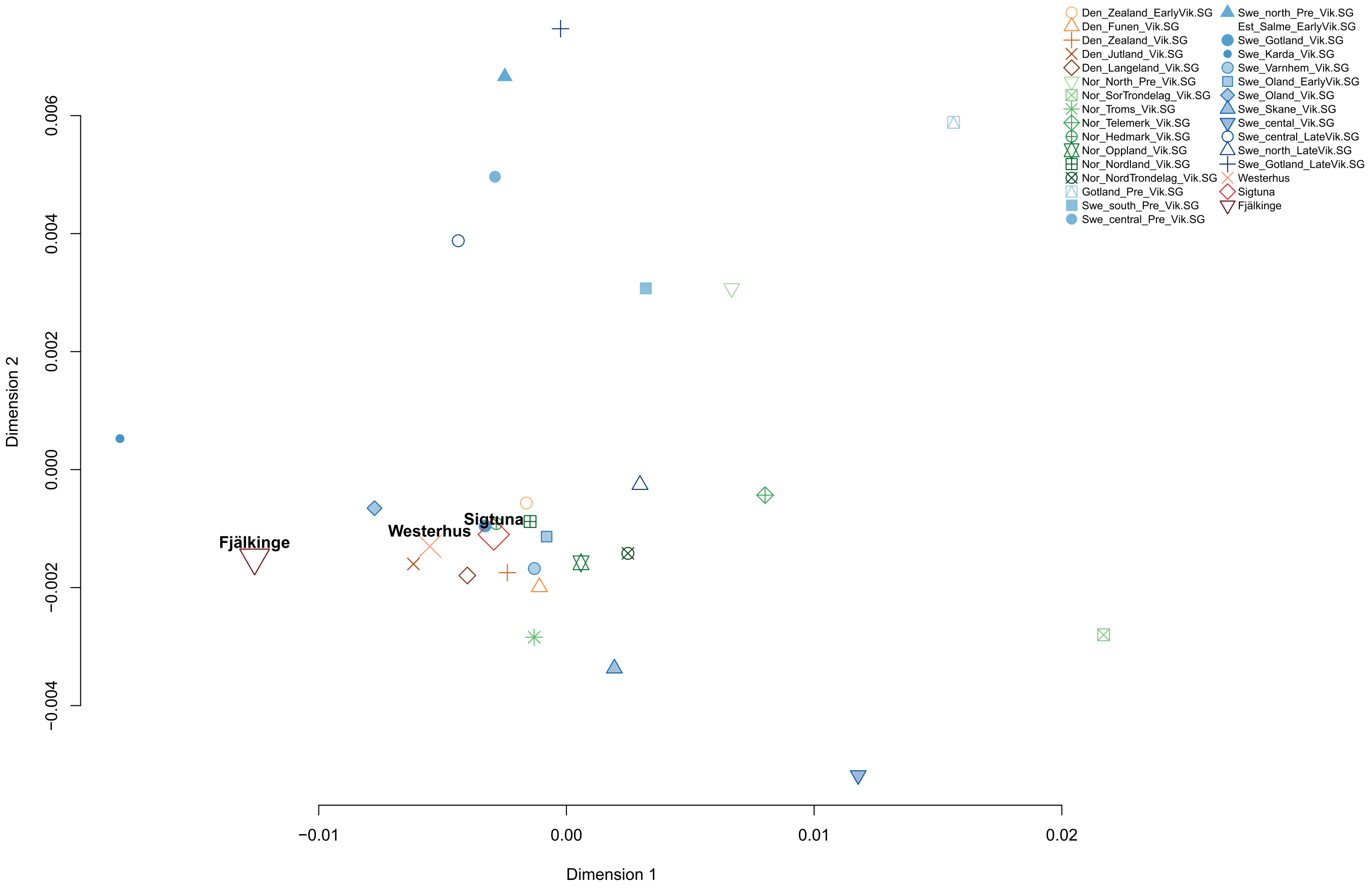


**Fig. S8.**

**MDS analysis of filtered population datasets.** MDS plot based on group f3 statistics for the further refined dataset. Population groups with fewer than five individuals were excluded, and from larger groups only the ten highest-coverage individuals were retained. This approach reduces the impact of sample-size imbalance and outliers, providing a clearer view of population-level structure.

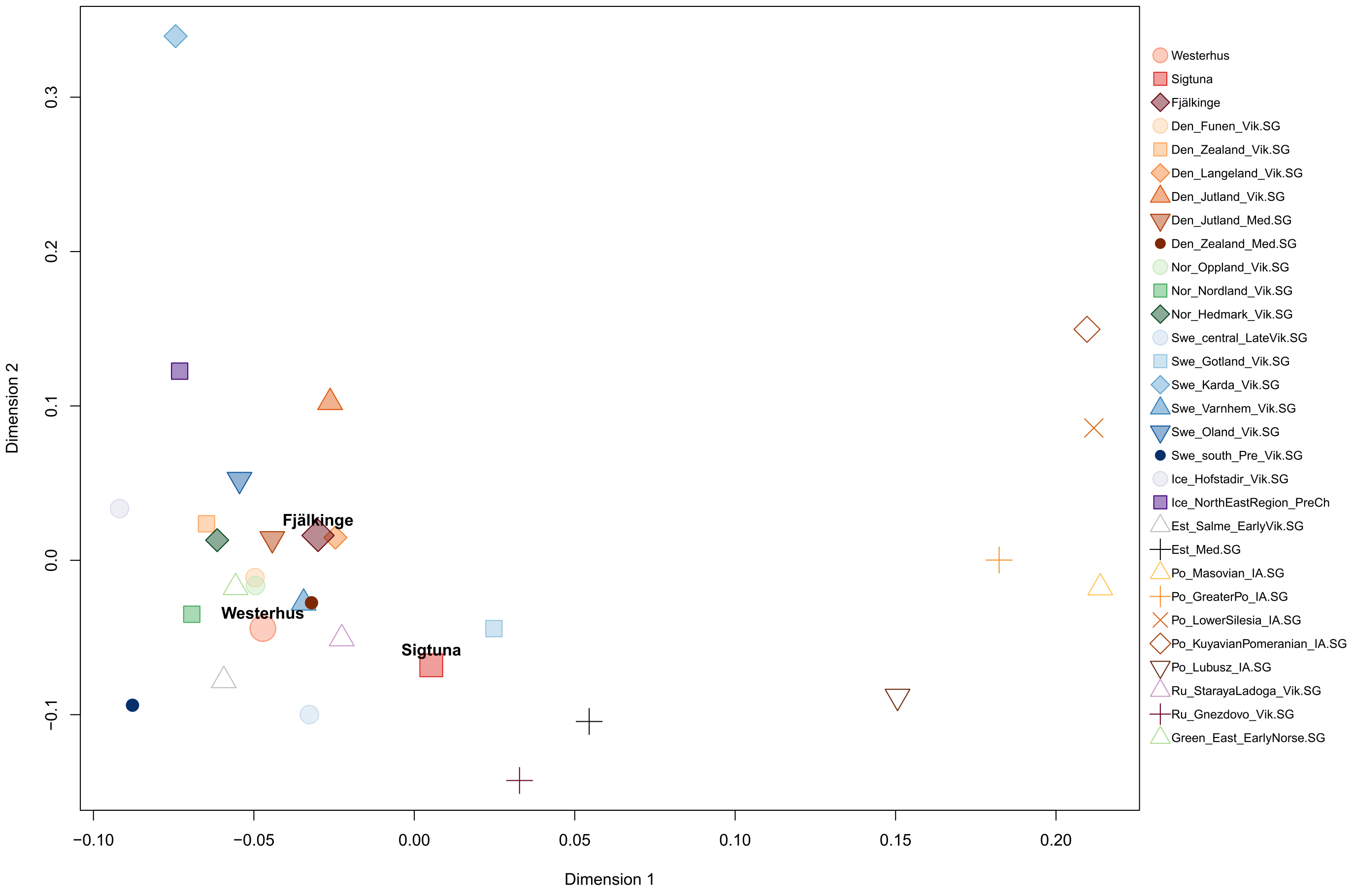


**Fig. S9.**
**Refined MDS analysis after outlier and sample-size correction.** MDS plot based on group f3 statistics for the further refined dataset. Population groups with fewer than five individuals were excluded, and from larger groups only the ten highest-coverage individuals were retained. This approach reduces the impact of sample-size imbalance and outliers, providing a clearer view of population-level structure.

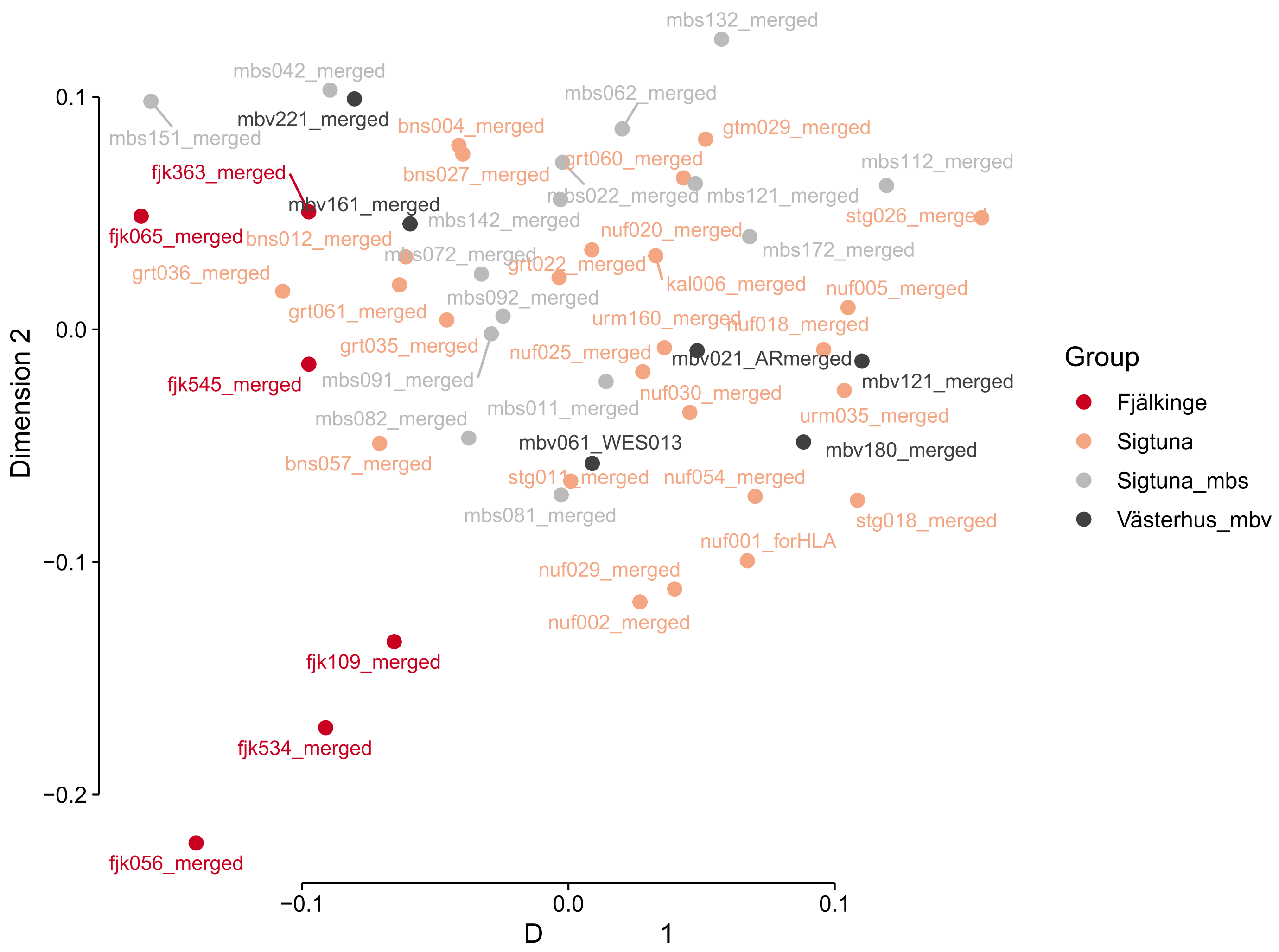


**Fig. S10.**
**Individual-level genetic distance scaling.** MDS plot based on group f3 statistics for 53 target individuals. A distance matrix was derived as 1-f3, and multidimensional scaling (MDS) was performed using classical scaling (cmdscale) in R (v. 4.4.3).

**Supplementary Tables**

| A | Site | Individuals in the group |
|---|---|---|
| Kindred 1 | Västerhus | **mbv080,** mbv111, mbv130, mbv172, mbv173, mbv190, mbv250, mbv251, mbv330, mbv332, mbv341, wes056, mbv160, mbv254, mbv291 |
| Kindred 2 | Västerhus | mbv240, mbv260, mbv321 |
| Kindred 3 | Västerhus | mbv070, mbv071, mbv124, mbv081 |
| Kindred 4 | Västerhus | mbv311, mbv331, mbv340 |
| Kindred 5 | Västerhus | **mbv080,** mbv114, mbv142, mbv253, mbv201 |
| Kindred 6 | Västerhus | mbv181, mbv290 |
| Kindred 7 | Fjälkinge | fjk531, fjk533 |
| Kindred 8 | Sigtuna | mbs161, mbs162 |
| Kindred 9 | Sigtuna | mbs021, mbs022 |
| Kindred 10 | Lund | win001, win002 |
| **B** | **Site** | **Individuals in the group** |
| Kindred 1, Kindred 3, Kindred 4, Kindred 5, Kindred 6 | Västerhus | wes007, mbv290, mbv130, mbv114, mbv142, mbv310, mbv341, mbv291, mbv252, mbv150, mbv251, mbv120, wes056, mbv201, mbv301, mbv123, mbv070, mbv181, mbv190, mbv191, WES003, mbv081, mbv270, mbv140, mbv253, mbv250, mbv254, mbv151, mbv131, mbv331, mbv111, mbv113, mbv160, mbv311, mbv040 |
| Kindred 7 | Fjälkinge | fjk531, fjk533 |
| Kindred 8 | Sigtuna | mbs161, mbs162 |
| Kindred 10 | Lund | win002, win001 |
| | Västerhus | WES005, mbv240 |
| | Sigtuna | mbs032, mbs031 |
| | Västerhus | mbv141, mbv281 |
| | Västerhus | WES001, mbv090 |

**Table S1.**

**Summary of pairwise relatedness and kindred groupings.** Individuals grouped according to identified pairwise relatedness where each of the individuals included in the group is linked with at least one other individual in the group. Relatedness was defined as: A) 1st or 2nd degree relatedness detected through kinship analysis. Please note individual mbv080 (grave 55a)

appears to be related both to individuals from kindred-1 and those from kindred-5. The two kindreds were kept separate in the pedigree reconstruction due to more than one possible topology (table S10); B) sharing of minimum at least three long IBD segments as identified through ancIBD (table S11).

**Data S1.** Supplementary Excel File. Contains Tables S2 – S15.

**Table S2**. Archaeological, osteological and genomic data information on all 165 samples used in this study (of which 142 are newly published).
**Table S3**. Summary statistics listing sex and age configuration in all included multiple burials.
**Table S4**. Datasets used in various analyses depending on relatedness status of the co-analysed individuals.
**Table S5.** READ results using transversions from EGDP.
**Table S6.** lcMLkin founders detected using transversions from 1000 Genome Project.
**Table S7.** lcMLkin all pairwise comparisons using transversions from 1000 Genome Project.
**Table S8.** NgsRelate results based on autosomal SNP selection from EGDP.
**Table S9**. NgsRelate results based on X chromosomal SNP selection from EGDP.
**Table S10.** Identified kinship - summary table.
**Table S11.** AncIBD results.
**Table S12.** HapROH results.
**Table S13**. Individual outgroup f3-statistics.
**Table S14**. Individual f4-statistics.
**Table S15.** Downsampled group-based outgroup f3-statistics.

## REFERENCES


1. L. Scheuer, S. M. Black, *Developmental Juvenile Osteology* (Academic Press, 2000).

2. J. E. Buikstra, D. H. Ubelaker, *Standards for Data Collection from Human Skeletal Remains* (Arkansas Archaeological Survey Research Series, 1994).

3. M. E. Lewis, *The Bioarchaeology of Children: Perspectives from Biological and Forensic Anthropology* (Cambridge Univ. Press, 2009).

4. T. K. Black III, Sexual dimorphism in the tooth-crown diameters of the deciduous teeth. *Am. J. Phys. Anthropol.* **48**, 77–82 (1978).

5. D. S. Weaver, Sex differences in the ilia of a known sex and age sample of fetal and infant skeletons. *Am. J. Phys. Anthropol.* **52**, 191–195 (1980).

6. H. Schutkowski, Sex determination of infant and juvenile skeletons: I. Morphognostic features. *Am. J. Phys. Anthropol.* **90**, 199–205 (1993).

7. A. Veroni, D. Nikitovic, M. A. Schillaci, Brief communication: Sexual dimorphism of the juvenile basicranium. *Am. J. Phys. Anthropol.* **141**, 147–151 (2010).

8. S. Thedéen, Who's that Girl? The cultural construction of girlhood and the transition to womanhood in Viking Age Gotland. *Child. Past* **1**, 78–93 (2008).

9. C. Hedenstierna-Jonson, "She came from another place: On the burial of a young girl in Birka (Bj463)," in *Viking Worlds: Things, Spaces and Movement*, M. Hem Erikse, U. Pedersen, B. Runderget, I. Axelsen, H. Lund Berg, Eds. (Oxbow Books, 2014), pp. 90–101.

10. P. Skoglund, J. Storå, A. Götherström, M. Jakobsson, Accurate sex identification of ancient human remains using DNA shotgun sequencing. *J. Archaeol. Sci.* **40**, 4477–4482 (2013).

11. K. Anastasiadou, M. Silva, T. Booth, L. Speidel, T. Audsley, C. Barrington, J. Buckberry, D. Fernandes, B. Ford, M. Gibson, A. Gilardet, I. Glocke, K. Keefe, M. Kelly, M. Masters, J. McCabe, L. McIntyre, P. Ponce, S. Rowland, J. Ruiz Ventura, P. Swali, F. Tait, D. Walker, H.

Webb, M. Williams, A. Witkin, M. Holst, L. Loe, I. Armit, R. Schulting, P. Skoglund, Detection of chromosomal aneuploidy in ancient genomes. *Commun. Biol.* **7**, 14 (2024).

12. N. A. Stewart, R. F. Gerlach, R. L. Gowland, K. J. Gron, J. Montgomery, Sex determination of human remains from peptides in tooth enamel. *Proc. Natl. Acad. Sci. U.S.A.* **114**, 13649–13654 (2017).

13. N. Price, Passing into poetry: Viking-Age mortuary drama and the origins of norse mythology. *Mediev. Archaeol.* **54**, 123–156 (2010).

14. A. Hed Jakobsson, J. Runer, A. Kjellström, T. Björk, *I Sigtunas utkant. Slutundersökningsrapport över gravar och bebyggelse vid Götes Mack* (Arkeologikonsult, 2017).

15. E. Naumann, M. Krzewińska, A. Götherström, G. Eriksson, Slaves as burial gifts in Viking Age Norway? Evidence from stable isotope and ancient DNA analyses. *J. Archaeol. Sci.* **41**, 533–540 (2014).

16. A. Kjellström, "The norm and the subaltern: Identifying slaves in an early medieval Scandinavian society," in *The Archaeology of Slavery in Early Medieval Northern Europe: The Invisible Commodity*, F. Biermann, M. Jankowiak, Eds. (Springer International Publishing, 2021), pp. 67–79.

17. A. Pedersen, "Grave, høje og kirker- gravskikke og monumenter mellom hedensk og kristet i Danmark," in *Från Hedniskt till Kristet. Förändringar i Begravningsbruk Och Gravskick i Skandinavien c:A 800–1200*, B. Nilsson, Ed. (Sällskapet Runica et Mediaevalia, 2010), pp. 57–102.

18. A.-S. Gräslunde, "Religionsskiftet speglat i gravskicket.: Ny svensk forskning kring senvikingatida gravar och gravskick," in *Från Hedniskt till Kristet. Förändringar i Begravningsbruk Och Gravskick i Skandinavien c:A 800–1200*, B. Nilsson, Ed. (Sällskapet Runica et Mediaevalia, 2010), pp. 131–164.

19. B. Nilsson, “Den tidiga medeltidens likbegängelse och begravningsbruk i Västkyrkan,” in *Från Hedniskt till Kristet. Förändringar i Begravningsbruk Och Gravskick i Skandinavien c:A 800–1200*, B. Nilsson, Ed. (Sällskapet Runica et Mediaevalia, 2010), pp. 9–56.

20. B. Nilsson, *Kvinnor, Män Och Barn På Medeltida Begravningsplatser: Women, Men and Children in Mediaeval Burial Places* (Lunne böcker, 1994).

21. S. Nordeide, ““I døden er vi alle ulike”: Gravmateriale i Sør-Norge i yngre jernalder og tidlig middelalder som grunnlag for å belyse kristningen av Norge,” in *Från Hedniskt till Kristet: Förändringar i Begravningsbruk Och Gravskick i Skandinavien ca. 800–1200*, B. Nilsson, Ed. (Sällskapet Runica et Mediavalia, 2010), pp. 102–130.

22. R. Gilchrist, “Transforming medieval beliefs. The significance of bodily resurrection to medieval burial rituals,” in *Death and Changing Rituals. Function and Meaning in Ancient Funerary Practices*, J. R. Brandt, H. Ingvaldsen, M. Prusac, Eds. (Oxbow Books, 2014), pp. 379–396.

23. A. Andrén, Ad sanctos – De dödas plats under medeltiden. *Hikuin* **27**, 7 (2000).

24. K. Jonsson, “Practices for the living and the dead: Medieval and post-Reformation burials in Scandinavia,” thesis, Stockholm University, Stockholm (2009).

25. N. G. Gejvall, *Westerhus: Medieval Population and Church in the Light of Skeletal Remains* (Kungl. Vitterhets-, historie- och antikvitetsakademien, 1960) Monografier 43.

26. J. Kieffer-Olsen, “Grav og gravskik i det middelalderlige Danmark: 8 kirkegårdsudgravninger,” thesis, Aarhus Universitet, Højbjerg (1993).

27. K. Jonsson, “Till döden skiljer oss åt…,” in *Västerhus: Kapell, kyrkogård och befolkning*, E. Iregren, V. Alexandersen, L. Redin, Eds. (Kungliga Vitterhets- historie- och antikvitetsakademien, 2009), pp. 40–63.

28. B. Nilsson, “Död och begravning. Begravningsskicket i Norden,” in *Tanke Och Tro: Aspekter På Medeltidens Tankevärld Och Fromhetsliv*, vol. 3 of *Studier till det medeltida Sverige*, O. Ferm, I. Estham, G. Tegnér, Eds. (Riksantikvarieämbetet, 1987), pp. 133–150.

29. B. Nilsson, "De sepulturis. Gravrätten i Corpus Iuris Canonici och medeltida nordisk lagstiftning," thesis, Stockholm University, Stockholm (1989).

30. M. Ranåker, "Flerpersonsgravar under medeltid. Västerhus kyrkogård belyst av andra begravningsplatser," in *Västerhus. Kapell, kyrkogård och befolkning.*, E. Iregren, V. Alexandersen, L. Redin, Eds. (Kungliga Vitterhets- historie- och antikvitetsakademien, 2009), pp. 26–39.

31. K. W. Alt, W. Vach, "Kinship studies in skeletal remains - Concepts and examples," in *Dental Anthropology: Fundamentals, Limits and Prospects*, K. W. Alt, F. W. Rösing, M. Teschler-Nicola, Eds. (Springer Vienna, 1998), pp. 537–554.

32. C. F. Ratican, *Bodies, Beings, and the Multiple Burial Rite of the Western Viking World* (Routledge, 2024).

33. K. M. Johnson, K. S. Paul, Bioarchaeology and kinship: Integrating theory, social relatedness, and biology in ancient family research. *J. Archaeol. Res.* **24**, 75–123 (2015).

34. A. Kjellström, "Tillsammans i evighet: Osteologiska aspekter på flerpersonsgravar i Sigtuna," in *Sigtuna Dei* (Sigtuna museer, 2010), pp. 127–136.

35. R. Rodríguez-Varela, K. H. S. Moore, S. S. Ebenesersdóttir, G. M. Kilinc, A. Kjellström, L. Papmehl-Dufay, C. Alfsdotter, B. Berglund, L. Alrawi, N. Kashuba, V. Sobrado, V. K. Lagerholm, E. Gilbert, G. L. Cavalleri, E. Hovig, I. Kockum, T. Olsson, L. Alfredsson, T. F. Hansen, T. Werge, A. R. Munters, C. Bernhardsson, B. Skar, A. Christophersen, G. Turner-Walker, S. Gopalakrishnan, E. Daskalaki, A. Omrak, P. Pérez-Ramallo, P. Skoglund, L. Girdland-Flink, F. Gunnarsson, C. Hedenstierna-Jonson, M. T. P. Gilbert, K. Lidén, M. Jakobsson, L. Einarsson, H. Victor, M. Krzewińska, T. Zachrisson, J. Storå, K. Stefánsson, A. Helgason, A. Götherström, The genetic history of Scandinavia from the Roman Iron Age to the present. *Cell* **186**, 32–46.e19 (2023).

36. R. E. Green, A. W. Briggs, J. Krause, K. Prüfer, H. A. Burbano, M. Siebauer, M. Lachmann, S. Pääbo, The Neandertal genome and ancient DNA authenticity. *EMBO J.* **28**, 2494–2502 (2009).

37. Q. Fu, H. Li, P. Moorjani, F. Jay, S. M. Slepchenko, A. A. Bondarev, P. L. F. Johnson, A. Aximu-Petri, K. Prüfer, C. de Filippo, M. Meyer, N. Zwyns, D. C. Salazar-García, Y. V. Kuzmin, S. G. Keates, P. A. Kosintsev, D. I. Razhev, M. P. Richards, N. V. Peristov, M. Lachmann, K. Douka, T. F. G. Higham, M. Slatkin, J.-J. Hublin, D. Reich, J. Kelso, T. B. Viola, S. Pääbo, Genome sequence of a 45,000-year-old modern human from western Siberia. *Nature* **514**, 445–449 (2014).

38. M. Rasmussen, X. Guo, Y. Wang, K. E. Lohmueller, S. Rasmussen, A. Albrechtsen, L. Skotte, S. Lindgreen, M. Metspalu, T. Jombart, T. Kivisild, W. Zhai, A. Eriksson, A. Manica, L. Orlando, F. M. De La Vega, S. Tridico, E. Metspalu, K. Nielsen, M. C. Ávila-Arcos, J. V. Moreno-Mayar, C. Muller, J. Dortch, M. T. P. Gilbert, O. Lund, A. Wesolowska, M. Karmin, L. A. Weinert, B. Wang, J. Li, S. Tai, F. Xiao, T. Hanihara, G. van Driem, A. R. Jha, F.-X. Ricaut, P. de Knijff, A. B. Migliano, I. G. Romero, K. Kristiansen, D. M. Lambert, S. Brunak, P. Forster, B. Brinkmann, O. Nehlich, M. Bunce, M. Richards, R. Gupta, C. D. Bustamante, A. Krogh, R. A. Foley, M. M. Lahr, F. Balloux, T. Sicheritz-Pontén, R. Villems, R. Nielsen, J. Wang, E. Willerslev, An aboriginal Australian genome reveals separate human dispersals into Asia. *Science* **334**, 94–98 (2011).

39. M. Krzewińska, R. Rodríguez-Varela, C. Ahlström Arcini, T. Ahlström, N. Hertzman, J. Storå, A. Götherström, Related in death? A curious case of a foetus hidden in bishop Peder Winstrup's coffin in Lund, Sweden. *J. Archaeol. Sci. Rep.* **37**, 102939 (2021).

40. M. Krzewińska, R. Rodríguez-Varela, R. Yaka, M. Vicente, G. Runfeldt, M. Sager, C. Ahlström Arcini, T. Ahlström, N. Hertzman, J. Storå, A. Götherström, Related in death? Further insights on the curious case of Bishop Peder Winstrup and his grandchild's burial. *Heritage* **7**, 576–584 (2024).

41. M. Krzewińska, A. Kjellström, T. Günther, C. Hedenstierna-Jonson, T. Zachrisson, A. Omrak, R. Yaka, G. M. Kılınç, M. Somel, V. Sobrado, J. Evans, C. Knipper, M. Jakobsson, J. Storå, A. Götherström, Genomic and strontium isotope variation reveal immigration patterns in a Viking Age Town. *Curr. Biol.* **28**, 2730–2738.e10 (2018).

42. A. Kjellström, *The Urban Farmer: Osteoarchaeological Analysis of Skeletons from Medieval Sigtuna Interpreted in a Socioeconomic Perspective* (Osteoarchaeological Research Laboratory, Stockholm Univ., 2005), theses and papers in Osteoarchaeology 2.

43. L. Mejsholm, "Constructions of early childhood at the syncretic cemetery of Fjälkinge: A case study," in *Youth and Age in the Medieval North* (Brill, 2008), pp. 37–56.

44. J. M. Monroy Kuhn, M. Jakobsson, T. Günther, Estimating genetic kin relationships in prehistoric populations. *PLOS ONE* **13**, e0195491 (2018).

45. M. Lipatov, K. Sanjeev, R. Patro, K. Veeramah, Maximum likelihood estimation of biological relatedness from low coverage sequencing data. bioRxiv 023374 [Preprint] (2015). https://doi.org/10.1101/023374.

46. K. Hanghøj, I. Moltke, P. A. Andersen, A. Manica, T. S. Korneliussen, Fast and accurate relatedness estimation from high-throughput sequencing data in the presence of inbreeding. *GigaScience* **8**, giz034 (2019).

47. T. S. Korneliussen, I. Moltke, NgsRelate: A software tool for estimating pairwise relatedness from next-generation sequencing data. *Bioinformatics* **31**, 4009–4011 (2015).

48. L. Pagani, D. J. Lawson, E. Jagoda, A. Mörseburg, A. Eriksson, M. Mitt, F. Clemente, G. Hudjashov, M. DeGiorgio, L. Saag, J. D. Wall, A. Cardona, R. Mägi, M. A. W. Sayres, S. Kaewert, C. Inchley, C. L. Scheib, M. Järve, M. Karmin, G. S. Jacobs, T. Antao, F. M. Iliescu, A. Kushniarevich, Q. Ayub, C. Tyler-Smith, Y. Xue, B. Yunusbayev, K. Tambets, C. B. Mallick, L. Saag, E. Pocheshkhova, G. Andriadze, C. Muller, M. C. Westaway, D. M. Lambert, G. Zoraqi, S. Turdikulova, D. Dalimova, Z. Sabitov, G. N. N. Sultana, J. Lachance, S. Tishkoff, K. Momynaliev, J. Isakova, L. D. Damba, M. Gubina, P. Nymadawa, I. Evseeva, L. Atramentova, O. Utevska, F.-X. Ricaut, N. Brucato, H. Sudoyo, T. Letellier, M. P. Cox, N. A. Barashkov, V. Škaro, L. Mulahasanovic, D. Primorac, H. Sahakyan, M. Mormina, C. A. Eichstaedt, D. V. Lichman, S. Abdullah, G. Chaubey, J. T. S. Wee, E. Mihailov, A. Karunas, S. Litvinov, R. Khusainova, N. Ekomasova, V. Akhmetova, I. Khidiyatova, D. Marjanović, L. Yepiskoposyan, D. M. Behar, E. Balanovska, A. Metspalu, M. Derenko, B. Malyarchuk, M. Voevoda, S. A. Fedorova, L. P. Osipova, M. M. Lahr, P. Gerbault, M. Leavesley, A. B.

Migliano, M. Petraglia, O. Balanovsky, E. K. Khusnutdinova, E. Metspalu, M. G. Thomas, A. Manica, R. Nielsen, R. Villems, E. Willerslev, T. Kivisild, M. Metspalu, Genomic analyses inform on migration events during the peopling of Eurasia. *Nature* **538**, 238–242 (2016).

49. P. A. Ewels, A. Peltzer, S. Fillinger, H. Patel, J. Alneberg, A. Wilm, M. U. Garcia, P. Di Tommaso, S. Nahnsen, The nf-core framework for community-curated bioinformatics pipelines. *Nat. Biotechnol.* **38**, 276–278 (2020).

50. A. Szolek, B. Schubert, C. Mohr, M. Sturm, M. Feldhahn, O. Kohlbacher, OptiType: Precision HLA typing from next-generation sequencing data. *Bioinformatics* **30**, 3310–3316 (2014).

51. A. G. Plascencia, M. Jakobsson, F. Sánchez-Quinto, Ancient DNA HLA typing reveals significant shifts in frequency in Europe since the Neolithic. *Sci. Rep.* **15**, 6161 (2025).

52. S. Rubinacci, D. M. Ribeiro, R. J. Hofmeister, O. Delaneau, Efficient phasing and imputation of low-coverage sequencing data using large reference panels. *Nat. Genet.* **53**, 120–126 (2021).

53. H. Ringbauer, Y. Huang, A. Akbari, S. Mallick, I. Olalde, N. Patterson, D. Reich, Accurate detection of identity-by-descent segments in human ancient DNA. *Nat. Genet. 56*, 143–151 (2023).

54. P. W. Hedrick, R. C. Lacy, Measuring relatedness between inbred individuals. *J. Hered.* **106**, 20–25 (2015).

55. H. Ringbauer, J. Novembre, M. Steinrücken, Parental relatedness through time revealed by runs of homozygosity in ancient DNA. *Nat. Commun.* **12**, 5425 (2021).

56. G. Miniaci, Multiple burials in ancient societies: Theory and methods from Egyptian archaeology. *Camb. Archaeol. J.* **29**, 287–307 (2019).

57. S. Crawford, G. Shepherd, "Children, childhood and society," in *IAA Interdisciplinary Series Vol. I: Studies in Archaeology, History, Literature and Art*, S. Crawford, G. Shepherd, Eds. (BAR International Series 1696, 2007), pp. 1–4.

58. C. A. Roberts, M. Cox, *Health & Disease in Britain: From Prehistory to the Present Day* (Sutton Publishing, 2003).

59. J. V. Sigurðsson, *Viking Friendship: The Social Bond in Iceland and Norway, c. 900–1300* (Cornell Univ. Press, 2017).

60. H. Vogt, *The Function of Kinship in Medieval Nordic Legislation* (Brill, ed. 1, 2010).

61. A. Magnúsdóttir, “Frillor och fruar. Politik och samlevnad på Island 1120–1400,” thesis, University of Gothenburg, Gothenburg (2001).

62. A. G. Magnúsdóttir, “Women and sexual politics,” in *The Viking World*, S. Brink, N. Price, Eds. (Routledge, 2008), pp. 40–48.

63. R. M. Karras, Concubinage and slavery in the Viking Age. *Scand. Stud.* **62**, 141–162 (1990).

64. T. Nors, Illegitimate children and their high-born mothers. *Scand. J. Hist.* **21**, 17–37 (1996).

65. A. Hansen, “Fosterage and dependency in medieval Iceland and its significance in Gísla Saga,” in *Youth and Age in Medieval North*, S. Lewis-Simpson, Ed. (Brill, 2008), pp. 73–86.

66. C. E. G. Amorim, S. Vai, C. Posth, A. Modi, I. Koncz, S. Hakenbeck, M. C. La Rocca, B. Mende, D. Bobo, W. Pohl, L. P. Baricco, E. Bedini, P. Francalacci, C. Giostra, T. Vida, D. Winger, U. von Freeden, S. Ghirotto, M. Lari, G. Barbujani, J. Krause, D. Caramelli, P. J. Geary, K. R. Veeramah, Understanding 6th-century barbarian social organization and migration through paleogenomics. *Nat. Commun.* **9**, 3547 (2018).

67. K. Wang, B. Tobias, D. Pany-Kucera, M. Berner, S. Eggers, G. A. Gnecchi-Ruscone, D. Zlámalová, J. Gretzinger, P. Ingrová, A. B. Rohrlach, J. Tuke, L. Traverso, P. Klostermann, R. Koger, R. Friedrich, K. Wiltschke-Schrotta, S. Kirchengast, S. Liccardo, S. Wabnitz, T. Vida, P. J. Geary, F. Daim, W. Pohl, J. Krause, Z. Hofmanová, Ancient DNA reveals reproductive barrier despite shared Avar-period culture. *Nature* **638**, 1007–1014 (2025).

68. Y. Tian, I. Koncz, S. Defant, C. Giostra, D. N. Vyas, A. Sołtysiak, L. Pejrani Baricco, R. Fetner, C. Posth, G. Brandt, E. Bedini, A. Modi, M. Lari, S. Vai, P. Francalacci, R. Fernandes,

A. Steinhof, W. Pohl, D. Caramelli, J. Krause, A. Izdebski, P. J. Geary, K. R. Veeramah, The role of emerging elites in the formation and development of communities after the fall of the Roman Empire. *Proc. Natl. Acad. Sci. U.S.A.* **121**, e2317868121 (2024).

69. A. Margaryan, D. J. Lawson, M. Sikora, F. Racimo, S. Rasmussen, I. Moltke, L. M. Cassidy, E. Jørsboe, A. Ingason, M. W. Pedersen, T. Korneliussen, H. Wilhelmson, M. M. Buś, P. de Barros Damgaard, R. Martiniano, G. Renaud, C. Bhérer, J. V. Moreno-Mayar, A. K. Fotakis, M. Allen, R. Allmäe, M. Molak, E. Cappellini, G. Scorrano, H. McColl, A. Buzhilova, A. Fox, A. Albrechtsen, B. Schütz, B. Skar, C. Arcini, C. Falys, C. H. Jonson, D. Błaszczyk, D. Pezhemsky, G. Turner-Walker, H. Gestsdóttir, I. Lundstrøm, I. Gustin, I. Mainland, I. Potekhina, I. M. Muntoni, J. Cheng, J. Stenderup, J. Ma, J. Gibson, J. Peets, J. Gustafsson, K. H. Iversen, L. Simpson, L. Strand, L. Loe, M. Sikora, M. Florek, M. Vretemark, M. Redknap, M. Bajka, T. Pushkina, M. Søvsø, N. Grigoreva, T. Christensen, O. Kastholm, O. Uldum, P. Favia, P. Holck, S. Sten, S. V. Arge, S. Ellingvåg, V. Moiseyev, W. Bogdanowicz, Y. Magnusson, L. Orlando, P. Pentz, M. D. Jessen, A. Pedersen, M. Collard, D. G. Bradley, M. L. Jørkov, J. Arneborg, N. Lynnerup, N. Price, M. T. P. Gilbert, M. E. Allentoft, J. Bill, S. M. Sindbæk, L. Hedeager, K. Kristiansen, R. Nielsen, T. Werge, E. Willerslev, Population genomics of the Viking world. *Nature* **585**, 390–396 (2020).

70. D. Castex, S. Kacki, Demographic patterns distinctive of epidemic cemeteries in archaeological samples. *Microbiol. Spectr.* **4**, 10.1128/microbiolspec.PoH-0015-2015 (2016).

71. M. Meyer, M. Kircher, Illumina sequencing library preparation for highly multiplexed target capture and sequencing. *Cold Spring Harb. Protoc.* **6**, pdb.prot5448 (2010).

72. M. Schubert, S. Lindgreen, L. Orlando, AdapterRemoval v2: Rapid adapter trimming, identification, and read merging. *BMC. Res. Notes* **9**, 88 (2016).

73. M. Martin, Cutadapt removes adapter sequences from high-throughput sequencing reads. *EMBnet. J.* **17**, 10.14806/ej.17.1.200 (2011).

74. T. Magoč, S. L. Salzberg, FLASH: Fast length adjustment of short reads to improve genome assemblies. *Bioinformatics* **27**, 2957–2963 (2011).

75. H. Li, R. Durbin, Fast and accurate long-read alignment with Burrows–Wheeler transform. *Bioinformatics* **26**, 589–595 (2010).

76. M. Kircher, "Analysis of high-throughput Ancient DNA sequencing data," in *Ancient DNA*, vol. 840 of *Methods in Molecular Biology*, B. Shapiro, M. Hofreiter, Eds. (Humana Press, 2012), pp. 197–228.

77. T. S. Korneliussen, A. Albrechtsen, R. Nielsen, ANGSD: Analysis of next generation sequencing data. *BMC Bioinformatics* **15**, 356 (2014).

78. H. Li, B. Handsaker, A. Wysoker, T. Fennell, J. Ruan, N. Homer, G. Marth, G. Abecasis, R. Durbin, 1000 Genomes Project Data Processing Subgroup, The sequence alignment/map format and SAMtools. *Bioinformatics* **25**, 2078–2079 (2009).

79. H. Weissensteiner, D. Pacher, A. Kloss-Brandstätter, L. Forer, G. Specht, H.-J. Bandelt, F. Kronenberg, A. Salas, S. Schönherr, HaploGrep 2: Mitochondrial haplogroup classification in the era of high-throughput sequencing. *Nucleic Acids Res.* **44**, W58–W63 (2016).

80. M. van Oven, A. Van Geystelen, M. Kayser, R. Decorte, M. H. D. Larmuseau, Seeing the wood for the trees: A minimal reference phylogeny for the human Y chromosome. *Hum. Mutat.* **35**, 187–191 (2014).

81. R. Martiniano, B. De Sanctis, P. Hallast, R. Durbin, Placing ancient DNA sequences into reference phylogenies. *Mol. Biol. Evol.* **39**, msac017 (2022).

82. S. Mallick, A. Micco, M. Mah, H. Ringbauer, I. Lazaridis, I. Olalde, N. Patterson, D. Reich, The Allen Ancient DNA Resource (AADR) a curated compendium of ancient human genomes. *Sci. Data* **11**, 182 (2024).

83. B. S. Weir, A. D. Anderson, A. B. Hepler, Genetic relatedness analysis: Modern data and new challenges. *Nat. Rev. Genet.* **7**, 771–780 (2006).

84.1000G enomes Project Consortium, G. R. Abecasis, A. Auton, L. D. Brooks, M. A. De Pristo, R. M. Durbin, R. E. Handsaker, H. M. Kang, G. T. Marth, G. A. McVean, An

integrated map of genetic variation from 1,092 human genomes. *Nature* **491**, 56–65 (2012).

85. M. Bastian, S. Heymann, M. Jacomy, Gephi: An open source software for exploring and manipulating networks. *Proc. Int. AAAI Conf. Web Soc. Media* **3**, 361–362 (2009).

86. T. M. J. Fruchterman, E. M. Reingold, Graph drawing by force-directed placement. *Softw Pract Exp* **21**, 1129–1164 (1991).

87. N. Patterson, A. L. Price, D. Reich, Population structure and eigenanalysis. *PLOS Genet.* **2**, e190 (2006).

88. N. Patterson, P. Moorjani, Y. Luo, S. Mallick, N. Rohland, Y. Zhan, T. Genschoreck, T. Webster, D. Reich, Ancient admixture in human history. *Genetics* **192**, 1065–1093 (2012).

89. F. L. Adair, R. E. Scammon, A study of the ossification centers of the wrist, knee and ankle at birth, with particular reference to the physical development and maturity of the newborn. *Am. J. Obstet. Gynecol.* **2**, 35–60 (1921).

90. L. Scheuer, S. M. Black, *The Juvenile Skeleton* (Elsevier Science, 2004).

91. J. Bruzek, A method for visual determination of sex, using the human hip bone. *Am. J. Phys. Anthropol.* **117**, 157–168 (2002).

92. V. Novotný, Sex determination of the pelvic bone: A systems approach. *Anthropologie* **24**, 197–206 (1986).

93. T. W. Phenice, A newly developed visual method of sexing the os pubis. *Am. J. Phys. Anthropol.* **30**, 297–301 (1969).

94. A. Kemkes-Grottenthaler, F. Löbig, F. Stock, Mandibular ramus flexure and gonial eversion as morphologic indicators of sex. *Homo* **53**, 97–111 (2002).

95. P. L. Walker, Sexing skulls using discriminant function analysis of visually assessed traits. *Am. J. Phys. Anthropol.* **136**, 39–50 (2008).

96. D. R. Hunt, Sex determination in the subadult ilia: An indirect test of Weaver's nonmetric sexing method. *J. Forensic Sci.* **35**, 881–885 (1990).

97. J. Irurita Olivares, I. Alemán Aguilera, Validation of the sex estimation method elaborated by Schutkowski in the Granada Osteological Collection of identified infant and young children: Analysis of the controversy between the different ways of analyzing and interpreting the results. *Int. J. Leg. Med.* **130**, 1623–1632 (2016).

98. D. M. Mittler, S. G. Sheridan, Sex determination in subadults using auricular surface morphology: A forensic science perspective. *J. Forensic Sci.* **37**, 1068–1075 (1992).

99. L. Scheuer, A blind test of mandibular morphology for sexing mandibles in the first few years of life. *Am. J. Phys. Anthropol.* **119**, 189–191 (2002).

100. R. C. Sutter, Nonmetric subadult skeletal sexing traits: I. A blind test of the accuracy of eight previously proposed methods using prehistoric known-sex mummies from northern Chile. *J. Forensic Sci.* **48**, 927–935 (2003).

101. S. Thedéen, "Immortal maidens: The visual significance of the colour white in girls' graves on Viking-Age Gotland," in *Making Sense of Things: Archaeologies of Sensory Perception. Stockholm Studies in Archaeology* (Department of Archaeology and Classical Studies, Stockholm University, 2010), vol. 53, pp. 103–120.

102. M. L. Stig Sørensen, *Gender Archaeology* (Polity Press, 2000).

103. M. Ivarsson-Aalders, M. Krzewińska, E. Karlsson, A. Götherström, A. Kjellström , Beyond the binary? A multi-method approach to sexing children at the Viking Age Site of Ihre, Gotland. *Int. J. Osteoarchaeol.* **35**, 233–247 (2025).

104. H. Guy, C. Masset, C.-A. Baud, Infant taphonomy. *Int. J. Osteoarchaeol.* **7**, 221–229 (1997).

105. S. E. Halcrow, N. Tayles, "The bioarchaeological investigation of children and childhood," in *Social Bioarchaeology*, vol 14 of *Blackwell Studies in Global Archaeology*, S. C. Agarwal, B. A. Glencross, Eds. (Blackwell Publishing Ltd., 2011), pp. 333–360.

106. S. Crawford, C. Lewis, Childhood studies and the society for the study of childhood in the past. *Child. Past* **1**, 5–16 (2008).

107. D. Pany-Kucera, K. Rebay-Salisbury, *Ages and Abilities: The Stages of Childhood and Their Social Recognition in Prehistoric Europe and Beyond* (Archaeopress, 2020).

108. C. E. Batey, C. Paterson, “A Viking burial at Balnakeil, Sutherland,” in *Early Medieval Art and Archaeology in the Northern World: Studies in Honour of James Graham-Campbell*, vol. 58 of Series: Northern World, A. Reynolds, L. Webster, Eds. (Brill, 2012), pp. 631–659.

109. C. Callow, “Transitions to Adulthood in Early Icelandic Society” in *Children, Childhood and Social Identity*, IAA Interdisciplinary Series, vol. 1 of *Studies in Archaeology, History, Literature and Art*, S. Crawford, G. Shepherd, Eds. (BAR International Series 1696, 2007), pp. 45–55.

110. P. G. Foote, D. M. Wilson, *The Viking Achievement: The Society and Culture of Early Medieval Scandinavia* (Sidgwick & Jackson, 1970).

111. J. Myrdal, G. Bäärnhielm, *Kvinnor, Barn & Fester i Medeltida Mirakelberättelser: Med En Katalog Över Svenska Mirakelberättelser Och En Nyöversättning Av Brynolfsmiraklerna* (Skaraborgs länsmuseum, 1994).

112. E. Iregren, Scandinavian women during the medieval period; health, childbirth and childcare. *Collegium Anthropologicum* **16**, 59–81 (1992).

113. N. Price, “Dying and the dead: Viking Age mortuary behaviour,” in *The Viking World* (Routledge, 2008), pp. 257–273.

114. A. M. Hållans Stenholm, *Fornminnen: Det Förflutnas Roll i Det Förkristna Och Kristna Mälardalen* (Nordic Academic Press, 2012), vol. 15.

115. I. Barbiera, Buried together, buried alone: Christian commemoration and kinship in the early Middle Ages. *Early Mediev. Eur.* **23**, 385–409 (2015).

116. P. Holck, *Cremated Bones: A Medical-Anthropological Study of an Archaeological Material on Cremation Burials* (Universitetet i Oslo, 1996).

117. C. Ekholst, “För varje brottsling ett straff: Föreställningar om kön i de svenska medeltidslagarna,” thesis, Historiska Institutionen, Stockholm (2009).

118. A. Ney, *Vänskap Mellan Kvinnor På Vikingatiden: Om Urval Och Historieskrivning i de Isländska Sagorna* (Nordic Academic Press, 2024).

119. E. Iregren, V. Alexandersen, H. Jungner, P. Isberg, “Diet and growth of medieval children in a rural society in Northern Europe,” in *Dieta y Crecimiento Infantil Medieval en una Sociedad Rural del Norte de Europa*, P. Montero, C. Prado, P. Acevedo Cantero, M. Carmenate, A. del Valle, J. Herrerín, J. F. Romero, K. Keller, N. López, A. I. Mora, Eds. (Sociedad Española de Antropología Física, 2015), pp. 391–406.

120. T. Zachrisson, C. Ljung, A. Kjellström, “Skärningspunkt Sigtuna: En första presentation av ett forskningsprojekt,” *Situne Dei: Årsskrift för Sigtunaforskning och historisk arkeologi* (Sigtuna Museum, 2017), pp. 52–63.

121. R. Edberg, “No kingdom without a town. Anund Olofsson’s policy for national independence and its materiality,” in *New Aspects on Viking-Age Urbanism c. AD 750–1100. Proceedings of the International Symposium at the Swedish History Museum, April 17th–20th 2013.*, L. Holmquist, S. Kalmring, C. Hedenstierna-Jonson, Eds. (Archaeological Research Laboratory, 2016), pp. 151–158.

122. J. Ros, “Sigtuna and the excavations at the Urmakaren and Trädgårdsmästaren sites,” in *New Aspects on Viking-Age Urbanism c. AD 750–1100: Proceedings of the International Symposium at the Swedish History Museum, April 17-20th 2013.*, L. Holmquist, S. Kalmring, C. Hedenstierna-Jonson, Eds. (Archaeological Research Laboratory, 2016), pp. 139–150.

123. J. Ros, *Sigtuna: The Town, the Churches and the Ecclesiastical Organisation*, vol. 30 of *Occasional Papers in Archaeology* (Uppsala Univ., 2001), 1100–6358.

124. S. Tesch, “Skilda gravar, skilda världar - tidigkristna gravar, kyrkor, stadsgårdar och storgårdar i Sigtuna och Mälarområdet,” in *Medeltida Storgårdar: 15 Uppsatser Om Ett Tvärvetenskapligt Forskningsproblem* (Gustav Adolfs Akademien, 2014), pp. 101–130.

125. M. Roslund, “Bridging two worlds: Tracing merchants from the Holy Roman Empire in high medieval Sigtuna,” in *Zwischen Fjorden Und Steppe: Festschrift Für Johan Callmer Zum 65. Geburtstag*, C. Theune, F. Biermann, R. Struwe, G. H. Jeut, Eds. (Verlag Marie Leidorf GmbH, 2010), pp. 239–250.

126. M. Roslund, “Crumbs from the rich man’s table: Byzantine finds in Lund and Sigtuna, c. 980–1250,” in *Visions of the Past: Trends and Traditions in Swedish Medieval Archaeology*, H. Andersson, L. Ersgård, P. Carelli, Eds. (Riksantikvarieämbete, 1997), pp. 239–297.

127. T. Zachrisson, “Sigtuna: An urban hub in the Viking world, and its roots,” in *Viking Encounters: Proceedings of the eighteenth Viking Congress, Denmark, August 6–12, 2017*, A. Pedersen, S. Sindbæk, Eds. (Oxbow Books Ltd., 2020), pp. 268–285.

128. T. Zachrisson, “Vikingatida Sigtuna, både nära och fjärran,” in *Tidens landskap : En vänbok till Anders Andrén*, C. Ljung, A. Andreasson Sjögren, I. Berg, E. Engström, A.-M. Hållans Stenholm, K. Jonsson, A. Klevnäs, L. Qviström, T. Zachrisson, Eds. (Nordic Academic Press, 2019), pp. 228–230.

129. S. Tesch, “Skiftet och Sigtuna: Hybriditet och motstånd som en del av Mälarområdets kristnande,” in *Skiftet: Vikingatida Sed Och Kristen Tro: Ett Mångvetenskapligt Perspektiv På Kristnadsprocessen i Mälarområdet* (Artos, 2017), pp. 11–52.

130. S. Tesche, “Sigtuna: Royal site and christian town and the regional perspective, c. 980–1100,” in *New Aspects on Viking-Age Urbanism c. AD 750–1100: Proceedings of the International Symposium at the Swedish History Museum, April 17-20th 2013.*, L. Holmquist, S. Kalmring, C. Hedenstierna-Jonson, Eds. (Archaeological Research Laboratory, Stockholm Univ., 2016), pp. 115–138.

131. M. Roslund, “A geography of slavery: Ceramic networks and communities in the Lake Mälaren valley, Sweden c. ad 950 to 1150,” in *Vikings Across Boundaries* (Routledge, 2020), pp. 258–284.

132. M. Roslund, “Tacit knowing of thralls: Style negotiation and hybridization among the unfree in 11th and 12th C. Sweden,” in *Archaeologies of Cultural Contact*, T. Clack, M. Brittain, Eds. (Oxford Univ. Press, 2022).

133. A. Kjellström, “Bioarchaeological Aspects of the Early Stage of Urbanization in Sigtuna, Sweden,” in *The Bioarchaeology of Urbanization: The Biological, Demographic, and Social Consequences of Living in Cities*, T. K. Betsinger, S. N. DeWitte, Eds. (Springer International Publishing, 2020), pp. 119–145.

134. C. Ljung, T. Zachrisson, A. Kjellström, På höjderna och i stadens utkant: Gravfält och gravgårdar i det äldsta Sigtuna. *Fornvännen* **2024**, 199–223 (2024).

135. M. M. Buś, M. Lembring, A. Kjellström, C. Strobl, B. Zimmermann, W. Parson, M. Allen, Mitochondrial DNA analysis of a Viking age mass grave in Sweden. *Forensic Sci. Int. Genet.* **42**, 268–274 (2019).

136. V. Alexandersen, E. Iregren, L. Redin, “Nya kunskaper om livet på storgården” in *Västerhus: Kapell, kyrkogård och befolkning*, E. Iregren, V. Alexandersen, L. Redin, Eds. (Kungliga Vitterhets- historie- och antikvitetsakademien, 2009), pp. 244–248.

137. E. Iregren, V. Alexandersen, L. Redin, *Västerhus: Kapell, Kyrkogård Och Befolkning* (Kungl. Vitterhets historie och antikvitets akademien, 2009).

138. V. Alexandersen, E. Iregren, Westerhus–Børnenes tænder. *Hikuin* **27**, 203 (2000).

139. T. Swärdstedt, *Odontological Aspects of a Medieval Population in the Province of Jämtland, Mid-Sweden* (University of Stockholm, 1966).

140. B. Helgesson, C. Arcini, A major burial ground fiscovered at Fjälkinge: Reflections of life in a Scanian Viking Village. *Lund Archaeol. Rev.* **2**, 51–61 (1996).

141. B. Helgesson, *Järnålderns Skåne: Samhälle, Centra Och Regioner* (Lund Univ., 2002), vol. 38.

142. O. Svensson, "Nämnda ting men glömda: Ortnamn, landskap och rättsutövning," thesis, Linnéuniversitetet, Växjö (2015).

143. C. Ahlström Arcini, T. D. Price, S. Hyll, *The Viking Age: A Time of Many Faces* (Oxbow Books, 2018).
